# Landscape evolution in China’s key ecological function zones during 1990–2015

**DOI:** 10.1038/s41598-024-52863-1

**Published:** 2024-02-01

**Authors:** Jiafeng Liu, Jing Zhong

**Affiliations:** 1 China Aero Geophysical Survey and Remote Sensing Center for Natural Resources, 267 North Fourth Ring Middle Road, Beijing, 100083 People’s Republic of China; 2 Key Laboratory of Digital Mapping and Land Information Application, Ministry of Natural Resources, 129 Luoyu Road, Wuhan, 430079 People’s Republic of China; 3 School of Resource and Environmental Sciences, Wuhan University, 129 Luoyu Road, Wuhan, 430079 People’s Republic of China

**Keywords:** Environmental impact, Evolutionary ecology

## Abstract

Landscape evolution has profound effects on ecosystems. Recently, some studies suggest that China has implemented plans leading in the greening of the world by mainly describing the changes based on satellite data. However, few studies have analyzed the policy effect on ecosystem improvement from the perspective of landscape pattern evolution. Among the numerous ecological policy plans, China’s key ecological function zones plan is an important one. In this study, we focus on depicting the long-term and large-scale landscape evolution in China’s key ecological function zones, which are accounting for 40.2% of China’s land area, and include four-type ecoregions where ecosystems are fragile or important, to comprehensively explore the environmental influences of policy planning. For this purpose, we first described the landscape composition changes and conversion mechanisms in China’s key ecological function zones from 1990 to 2015. Then we captured the detailed pattern evolution characteristics by landscape indices. The results show that these ecoregions were mostly evolving in an unfavorable direction in these 25 years, i.e. destruction of habitats and increment of fragmentation. Although greening areas increased based on other recent researches, the landscape pattern became worse, indicating it is necessary for the detailed analysis of landscape ecology and more accurate ecological planning. We also found the deterioration of the ecological environment had been uncharacteristically stopped or even improved in wind prevention and sand fixation ecoregions and biodiversity maintenance ecoregions after the implementation of this plan. Furthermore, we assumed that the policy is more prominent in these prohibiting sabotages and protecting areas with fragile ecological bases, which may be caused by the differentiated transfer payments in different ecoregions. Finally, some planning suggestions, such as stricter land use control, the regional balance of ecological transfer payments and deepening of ecological migration policies, etc., were proposed for promoting better future environmental changes.

## Introduction

In the worldwide landscape evolutions, the ecological environment has been severely damaged, resulting in a series of ecological problems such as habitat losses, climate changes, and reductions of biodiversity^1–7^. These landscape evolutions may be caused by deforestation, water loss, cropland and urban expansion, which is to supply the resources needed for human interests^8–13^. Several recent researches revealed that the important ecological landscape of forest in the world has increased significantly based on satellite data, and China have made considerable contributions to such increases^14–17^.

China's ecological environment has been receiving global attention, mainly because of its diversity and complexity, as well as its profound impact on global ecological environment^18–20^. China has also taken many actions to protect and enhance the particular ecological environment^16,21–26^. Among them, China’s key ecological function zones (hereinafter referred to as KEZs) based on four-type ecoregions (see section “Study area and data”) is also an important measure aiming to implement ecological sustainable development, through ecological investment and regional plan for protecting and improving the ecological environment^25^. Numerous countries regard ecologically protected areas as an effective way of protecting the ecological environment in ecoregions^27,28^. The ecologically protected areas are basically built in the ecoregions with particular ecological characteristics^29–32^. An ecoregion can be defined as a region with relatively homogeneous ecosystems at any spatial scales^33^. Ecoregions are thus constantly changing in terms of the biotic and abiotic ecosystem characteristics^34^. The landscape evolutions of KEZs, which are accounting for 40.2% of China’s land area and include four-type ecoregions with different ecological features. Nevertheless, existing researches focused on deductively depicting the amount (or area) changes in a single landscape, while lacking a description of the evolutionary characteristics of the spatial patterns of the landscape.

As ecological landscape area reduction is harmful to the ecosystem, habitat fragmentation is also harmful in terms of ecosystem services^3,4,6,35–39^. Habitat fragmentation is generally identified as the process of breaking apart of habitat with roughly following spatial characteristics: (a) reduction in fragment area, (b) increase in isolation, and (c) increase in exposed edges of habitat patches^40–42^. In the past, many researchers assumed both effects of habitat loss and fragmentation are confusing because of past nonmanipulative studies, and even multiple simultaneous effects over long time scales of fragmentation per se are unclear^43–45^. However, through several long-term repeatable experiments by controlling for confounding factors, impacts of habitat fragmentation are proved to be strong and typically degrading on biodiversity and ecological processes independent of habitat loss^46^. In detail, reduced fragment area, increased isolation, and increased edge these three aspects of fragmentation have detrimental effects on diverse ecological functions^4^. Therefore, it is desirable to depict the landscape pattern evolution with both area and pattern for comprehensively understanding the ecological environment changes.

The description of the landscape pattern evolution can be done using the landscape indices^47–50^. Most landscape indices are derived from statistical theory, information theory and fractal geometry^51–53^. Moreover, landscape indices have been widely applied for depicting the ecological process by measuring landscape composition and configuration^54,55^. Some of them have excellent performance in measuring spatial characteristics, i.e. area, edge and spatial relationship between patches, which can be used to reflect the aspects of fragmentation process.

In summary, it is important to reveal for comprehensively understanding the change characteristics of environment and exploring whether policy plan will better transform ecosystems in KEZs. In this study, we adopt verified landscape indices to depict the long-term and large-scale landscape evolution characteristics in KEZs from 1990 to 2015. Thus, we discussed the question of whether the ecological pattern is really getting better in the context of China becoming green. Moreover, the ecological impact of KEZs plan is discussed to reveal the effects of policy planning.

## Study area and data

**Figure 1:**
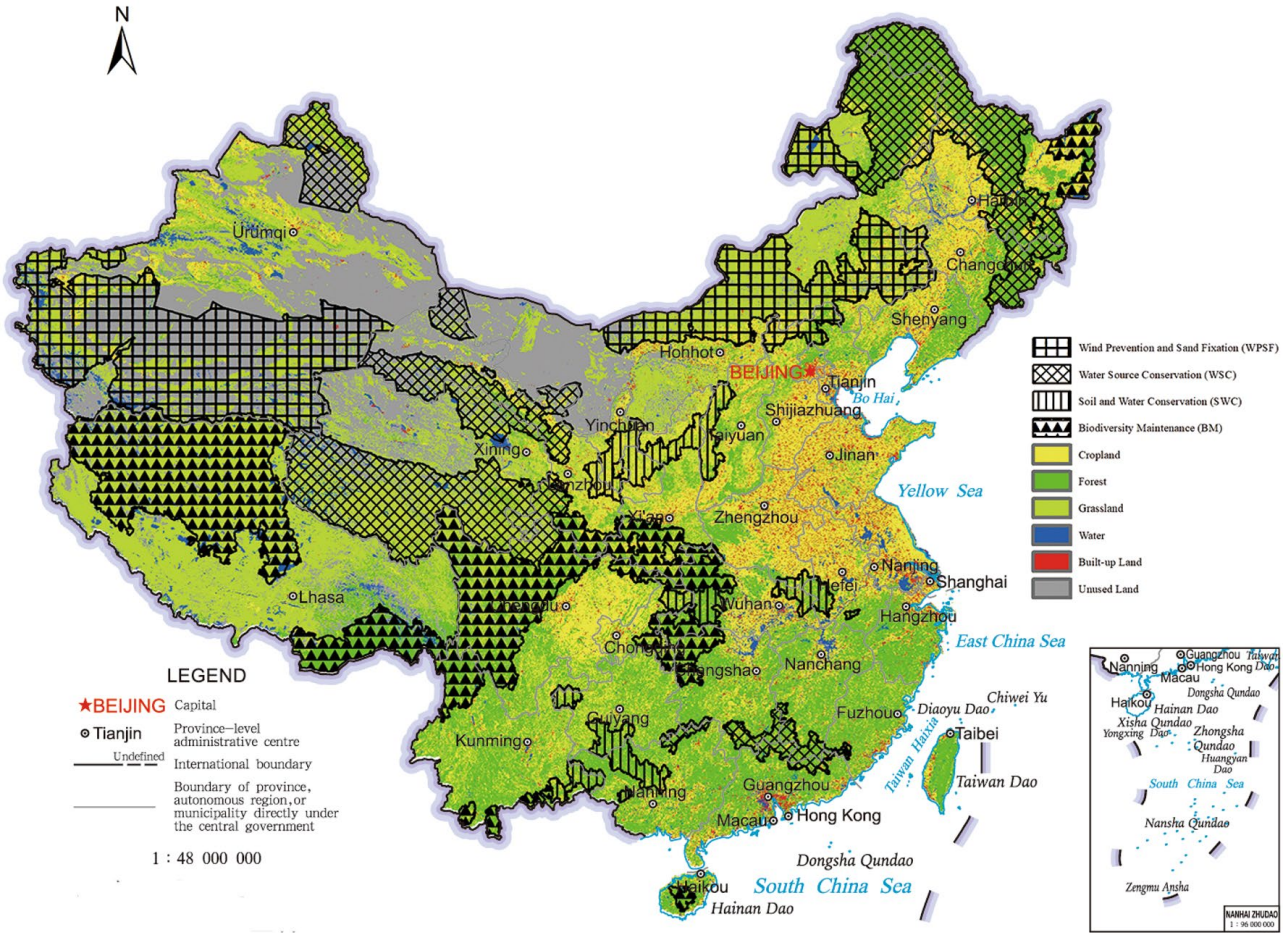
The position and landscape classes of four-type ecoregions in KEZs. The four ecoregions, which are made up of county-level administrative districts^25^, are shown in different marks. The map was created in ArcMap 10.7 (www.arcgis.com). The base map was supervised by the Ministry of Natural Resources of the People’s Republic of China, with review map number is GS (2019) 1673. Land use data for China from 1990 to 2015 were provided by Data Center for Resources and Environmental Sciences, Chinese Academy of Sciences^56^.

KEZs consist of 436 county-level administrative districts with a total area accounting for 40.2% of China’s land area as shown in Fig. 1^25^. The KEZs protect the regional ecological environment by adapting to the local ecological characteristics of four- type ecoregions, e.g. wind prevention and sand fixation ecoregions (hereinafter referred to as WPSFs), biodiversity maintenance ecoregions (hereinafter referred to as BMs), soil and water conservation ecoregions (hereinafter referred to as SWCs) and water source conservation ecoregions (hereinafter referred to as WSCs). The WPSFs refer to ecoregions with high sensitivity to serious desertification and frequent occurrence of sandstorms, mainly located in the northern part of China. The BMs refer to the ecoregions as major habitats of endangered rare animals and plants, mainly located in the southwest of China. The SWCs refer to the ecoregions need maintain water and soil functions because of serious soil erosion, mainly located in South Central China. The WSCs refer to the ecoregions as sources of important rivers and important water supply areas in China, mainly located in the northeast, central and southeastern parts of China.

The land use maps of China in 1990, 2000, 2005, 2010 and 2015 were provided by Data Center for Resources and Environmental Sciences, Chinese Academy of Sciences^54^. The original classification of land use types includes 6 first-level types (i.e., forest, grassland, cropland, water, built-up land and unused land). Among them, the unused land includes sandy land, Gobi, salina, bare land, alpine desert and tundra. Moreover, the forest and grassland are considered as major habitats for animals and plants, and the cropland is generally considered as a hospitable matrix. In this study, we employed the 6 landscape types and divided five time points into four periods, i.e. T1 (1990–2000), T2 (2000–2005), T3 (2005–2010) and T4 (2010–2015), to evaluate evolution process in landscape patterns.

## Methodology

As the landscape indices has been shown to be an effective measure of landscape fragmentation, we selects the landscape indices to describe the spatial characteristics of the landscape pattern evolution for KEZs^46,57,58^.

In this study, we use the type-level indices, because landscape-level indices contain information which is not crucial to the habitat class-level specific response variables^59^. When selecting the landscape indices, we also considered the following three principles: (1) widely used and recognized; (2) relatively independent of each other to avoid confusion; and (3) easy to calculated, so that it can be used in KEZs with large areas. Finally, in addition to landscape amount (class area, CA), we selected seven indices based on three aspects of fragmentation, i.e., (1) patch size: the largest patch index (LPI) and mean patch area (MN_AREA), (2) isolation: number of patches (NP) and patch cohesion index (COHESION), (3) edge: total edge (TE) and area-weighted mean shape index (AM_SHAPE). Moreover, a comprehensive index of reflecting the fragmentation, i.e., effective mesh size (MESH) is also be selected. In particular, the MESH, representing an intensive and area-proportionately additive measure, proves to be well suited for comparing the fragmentation of regions with differing total size^60^. The detailed explanations of all eight landscape metrics are shown in Table 1.

**Table 1:** Detailed explanations of eight landscape metrics.

Metrics (Abbreviation)	Formula	Description
Total class area (CA)	${\text{CA = }}\sum\limits_{j = 1}^{{\text{n}}} {a_{ij} \left( \frac{1}{1000000} \right)}$	*i* means the landscape class *i*. *a*_*ij*_ is area (m^2^) of patch *j* in *i*. *n* is total number of patches in *i*. CA equals the sum of the areas (m^2^) of all patches, divided by 10,000 (to convert to square kilometre); that is, total class area. Class area is a measure of landscape composition
Largest patch index (LPI)	$LPI = \frac{{\max_{j = 1}^{n} (a_{ij} )}}{A}(100)$	*A* is total landscape area (m^2^). LPI at the class level quantifies the percentage of total landscape area comprised by the largest patch. As such, it is a simple measure of dominance
Mean area (MN_AREA)	$MN\_AREA = \frac{{\sum\nolimits_{j = 1}^{n} {a_{ij} } }}{{10000n_{j} }}$	MN_AREA equals the mean area of the patches in landscape *i*, divided by 10,000 (to convert to hectares, software default). Mean area is a measure of patch scale in landscape *i* combining the information of class area and patches number
Number of patches (NP)	$NP = n_{i}$	*n*_*i*_ is number of patches in* i*. NP equals the number of patches of the corresponding landscape class. Number of patches type is a simple measure of the extent of subdivision or fragmentation of the patch type
Patch cohesion index (COHESION)	$COHESION = 100\left[ {1 - \frac{{\sum\nolimits_{j = 1}^{n} {e_{ij} } }}{{\sum\nolimits_{j = 1}^{n} {e_{ij} \sqrt {a_{ij} } } }}} \right]\left[ {1 - \frac{1}{\sqrt Z }} \right]^{ - 1}$	*Z* is the total number of grids in the landscape *i*. 0 < COHESION < 100. COHESION approaches 0 as the proportion of the landscape comprised of the focal class decreases and becomes increasingly subdivided and less physically connected. COHESION increases monotonically as the proportion of the landscape comprised of the focal class increases until an asymptote is reached near the percolation threshold
Total edge (TE)	$TE = \sum\limits_{j = 1}^{n} {e_{ij} }$	*e*_*ij*_ is total length (m) of edge in landscape *i*. TE equals the sum of the lengths (m) of all edge segments involving the corresponding landscape type
Area-weighted Mean Shape Index (AM_SHAPE)	$AM\_SHAPE = \sum\nolimits_{j = 1}^{n} {\left[ {\frac{{0.25e_{ij} }}{{\sqrt {a_{ij} } }}\left( {\frac{{a_{ij} }}{{\sum\nolimits_{j = 1}^{n} {a_{ij} } }}} \right)} \right]}$	Shape index corrects for the size problem of the perimeter-area ratio index by adjusting for a square standard and, as a result, is the simplest and perhaps most straightforward measure of shape complexity. Area-weighted mean shape index also takes into account the impact of different patch size
Effective mesh size (MESH)	$MESH = \frac{{\sum\nolimits_{j = 1}^{n} {a_{ij}^{2} } }}{10000A}$	MESH equals the sum of patch area squared, summed across all patches of the corresponding patch type, divided by the total landscape area (m2), divided by 10,000 (to convert to hectares, software default). The area squared of all patches in landscape *i* divided by the entire landscape area, describing landscape fragmentation. Higher values of MESH represent a lower degree of fragmentation

All of the above eight indices are already widely used and recognized in various regions in measuring fragmentation^61–64^. Then, the selected indices have been confirmed to better describe the landscape pattern characteristics with low redundancy^57,59^. In practice, the spatial pattern analysis program “Fragstats” provides a good help to calculate the landscape indices in KEZs with large areas^48^.

## Results

### The changes of landscape area in KEZs

**Figure 2:**
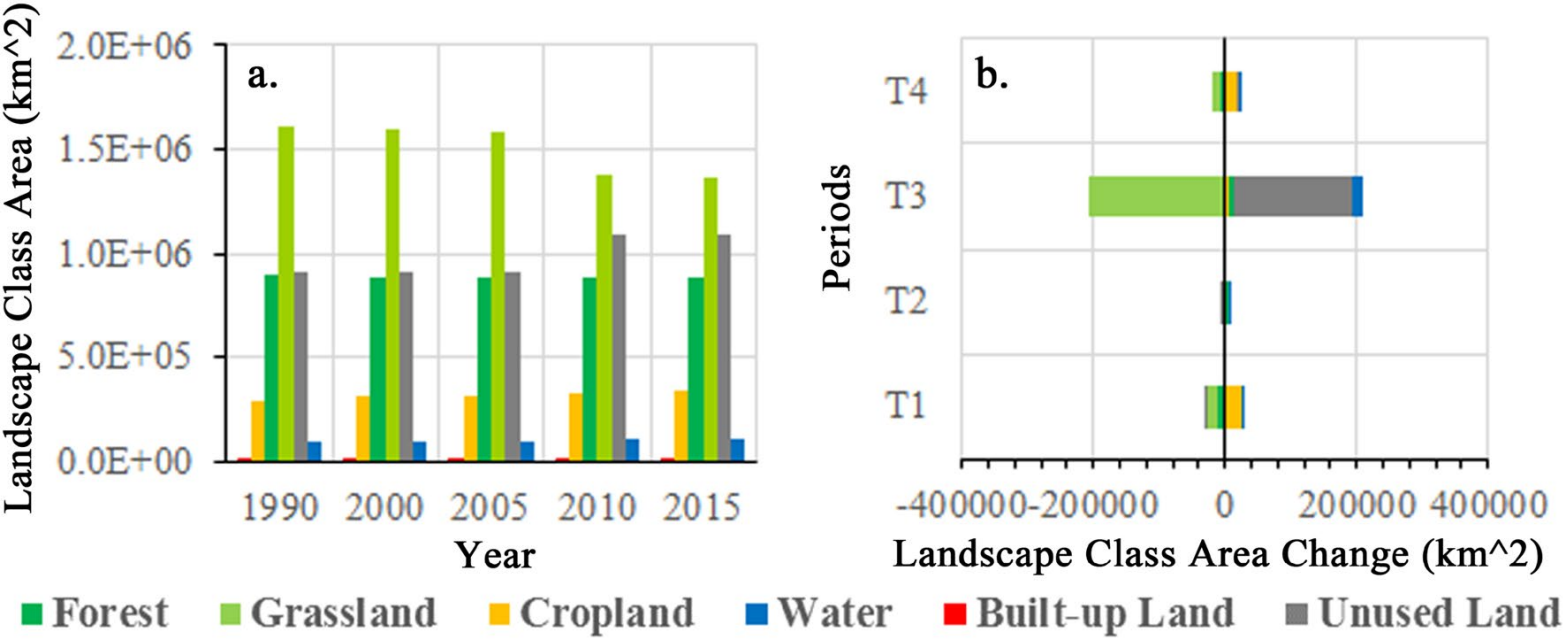
Entire KEZs landscape areas and its changes in different periods.

The study analyzed the changes in landscape areas in KEZs during the period of 1990–2015 to gain insight into the ecological environment. The analysis revealed that grassland, forest, and unused land were the dominant landscape classes in all five time points, while cropland, water, and built-up land were less prevalent. This suggests that the KEZs are mainly comprised of beneficial ecological landscapes, such as habitats, but also contain a significant amount of unused land, which may result in a fragile ecological environment. Detailed information on the landscape class areas and their distribution is provided in Fig. 2a and Table 1 (Appendix).

The areas of different landscape classes were changing with unequal characteristics. During the study periods, the amount of landscapes growths and the corresponding reductions are shown in Fig. 2b. In the T1 period, the areas of forest, grassland and unused land were decreased whereas cropland and water were increased. Relative to the negligible landscape changes during the T2 period, the changes of the T3 were huge. In the T3 period, most landscape classes had increased, but a huge amount of grassland disappeared. In the T4 period, the main change was the reduction of habitats, e.g. grassland and forest, accompanied by an increment in cropland.

Thus, the largest change in landscape area occurred in the T3 period, while landscape area changes were smaller in other periods. The main changes of landscape areas were reduction of grassland, and growth of unused land. The general reduction of grassland with increase of cropland and unused land indicates that the ecological environment of entire protection areas were frangible especially for the grassland.

**Figure 3:**
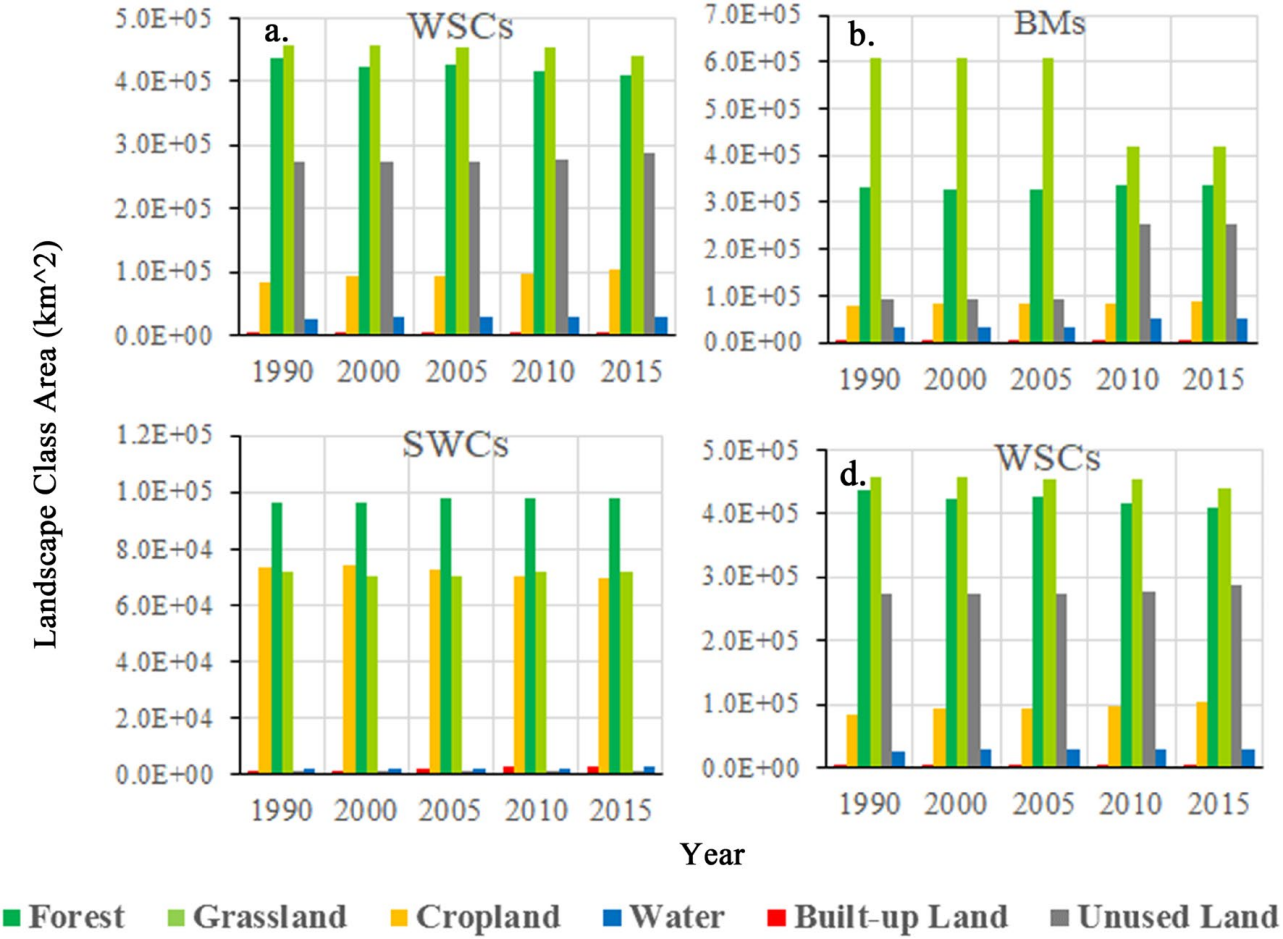
The landscape areas of four-type ecoregions in KEZs in five time point.

Since the four ecoregions of EFZs have different ecological features, we should depict their landscape area evolutions in depth for protecting and exerting their ecological function reasonably. The landscape compositions of four type ecoregions in 1990–2015 are represented in Fig. 3 and Tables 2–5 (Appendix). There are differences in the main landscapes in each ecoregion as shown below: (a) grassland and unused land in WPSFs, (b) grassland and forest in BMs, (c) forest, grassland and cropland in SWCs, and (d) grassland, forest and unused land in WSCs. The habitats, i.e. forests and grasslands, are the main landscape components in all ecoregions except WPSFs. These results indicate that the ecological landscape of WPSFs is fragile. Moreover, the grassland of BMs had been severely damaged during the T3 period while other ecoregions seemed to have little changed.

**Figure 4:**
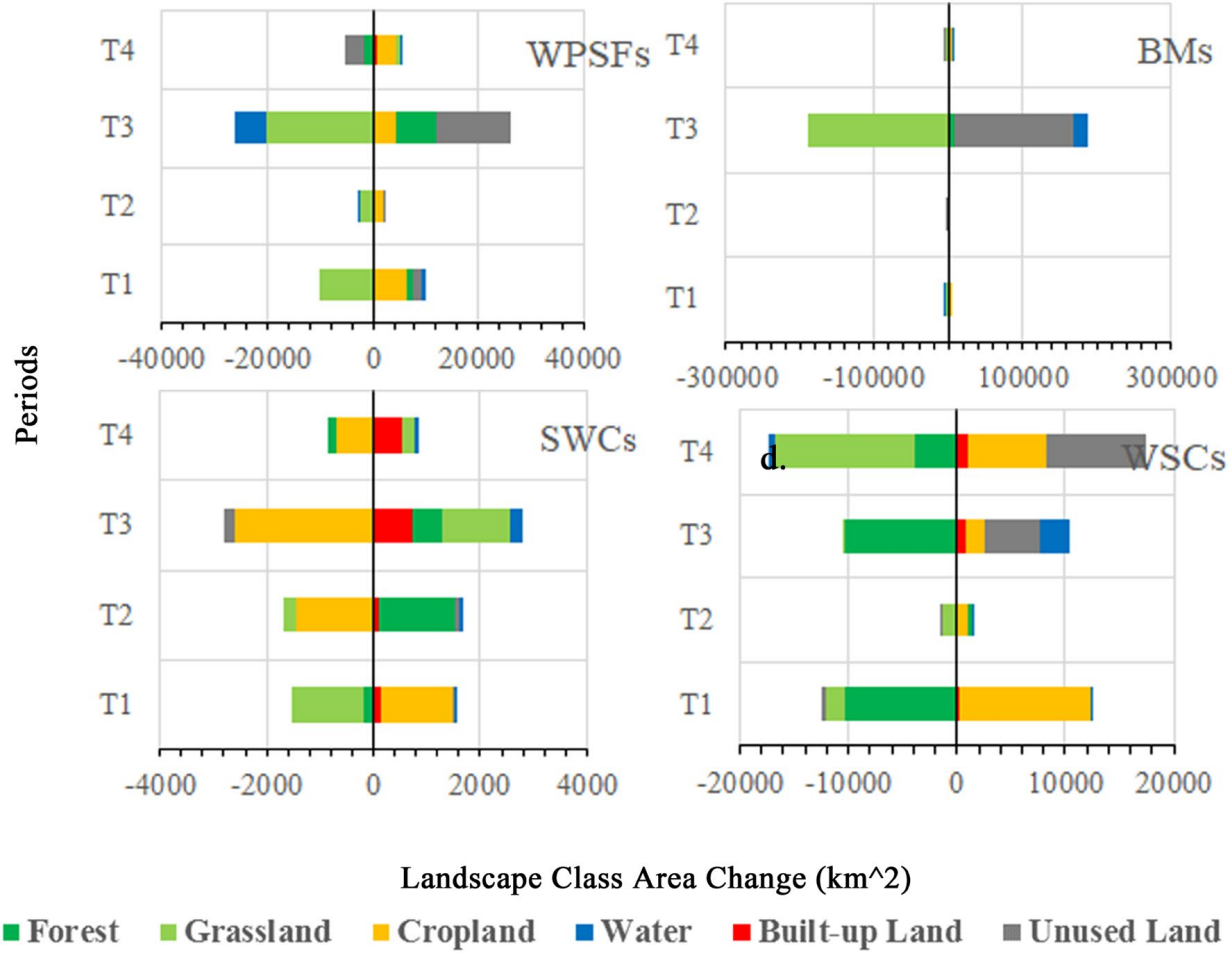
The landscape area changes of four-type ecoregions in KEZs during four periods.

The detail landscape area changes in the four ecoregions are shown with Fig. 4. In WPSFs, the landscape area changes mainly occurred in the T1 and T3 periods. Grassland was constantly decreased, while another main landscape unused land had a tortuous growth. The cropland was increased in all the four periods and had the largest increase among all the six landscapes. Besides, the increase of forest occupied in T1 and T3. In BMs, landscape area changes mainly happened in the T3. This was mainly caused by the reduction of the grassland and the increase of unused land. There was still a small increase of water and forest. In SWCs, the forest was largely increased in the T2 and T3, while the grassland was extensively decreased in T1 but increased in T3. The cropland was declined in all periods except for the T1. In WSCs, the landscape area changes were significantly occurred except for the T2. The dominant landscape classes, grassland and forest were constantly decreased. Conversely, the cropland was increased in all four periods, while the unused land was evidently increased in the T3 and T4.

### Landscape transformation characteristics in KEZs

**Figure 5:**
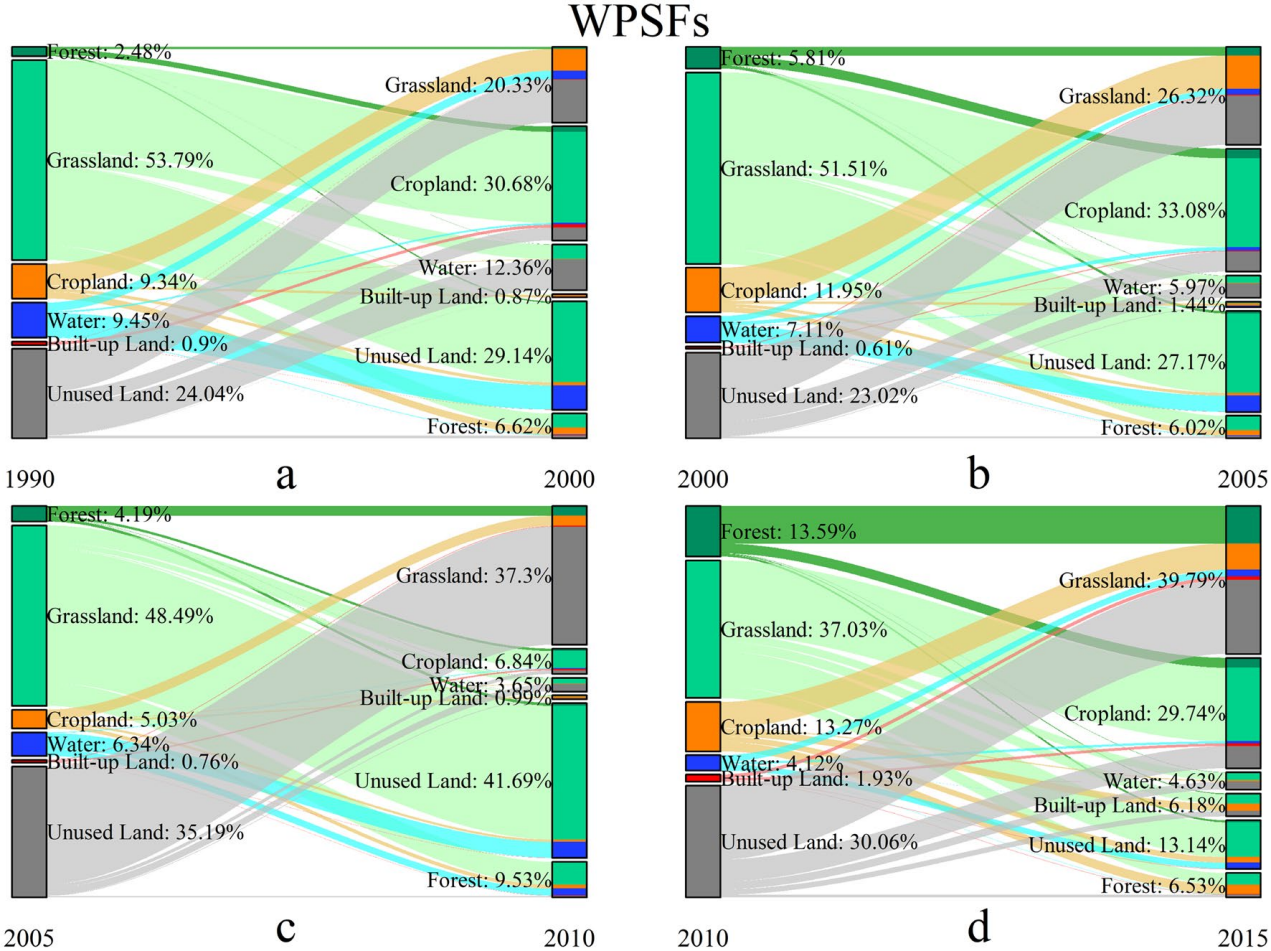
The landscape transformation in WPSFs during 1990–2015.

**Figure 6:**
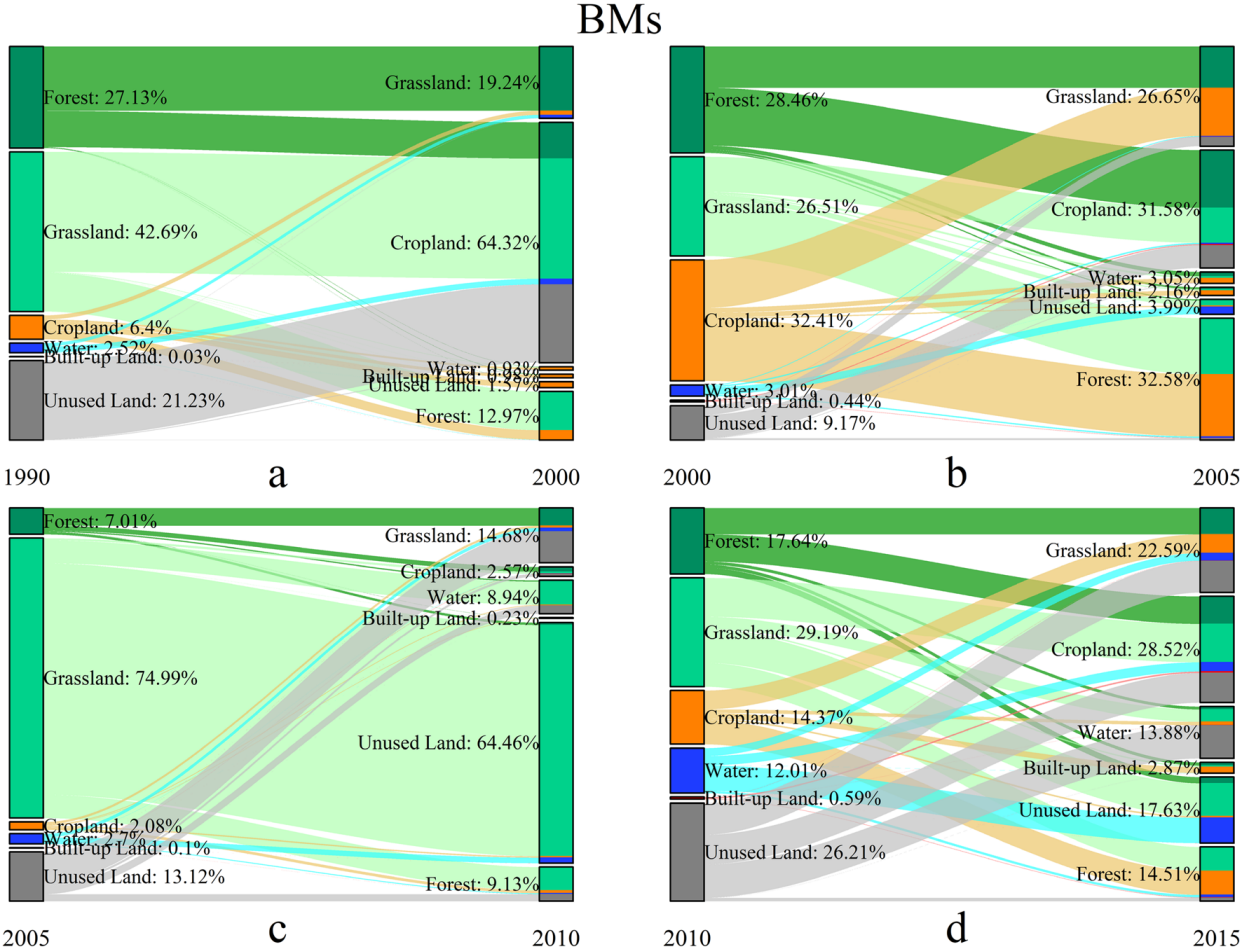
The landscape transformation in BMs during 1990–2015.

**Figure 7:**
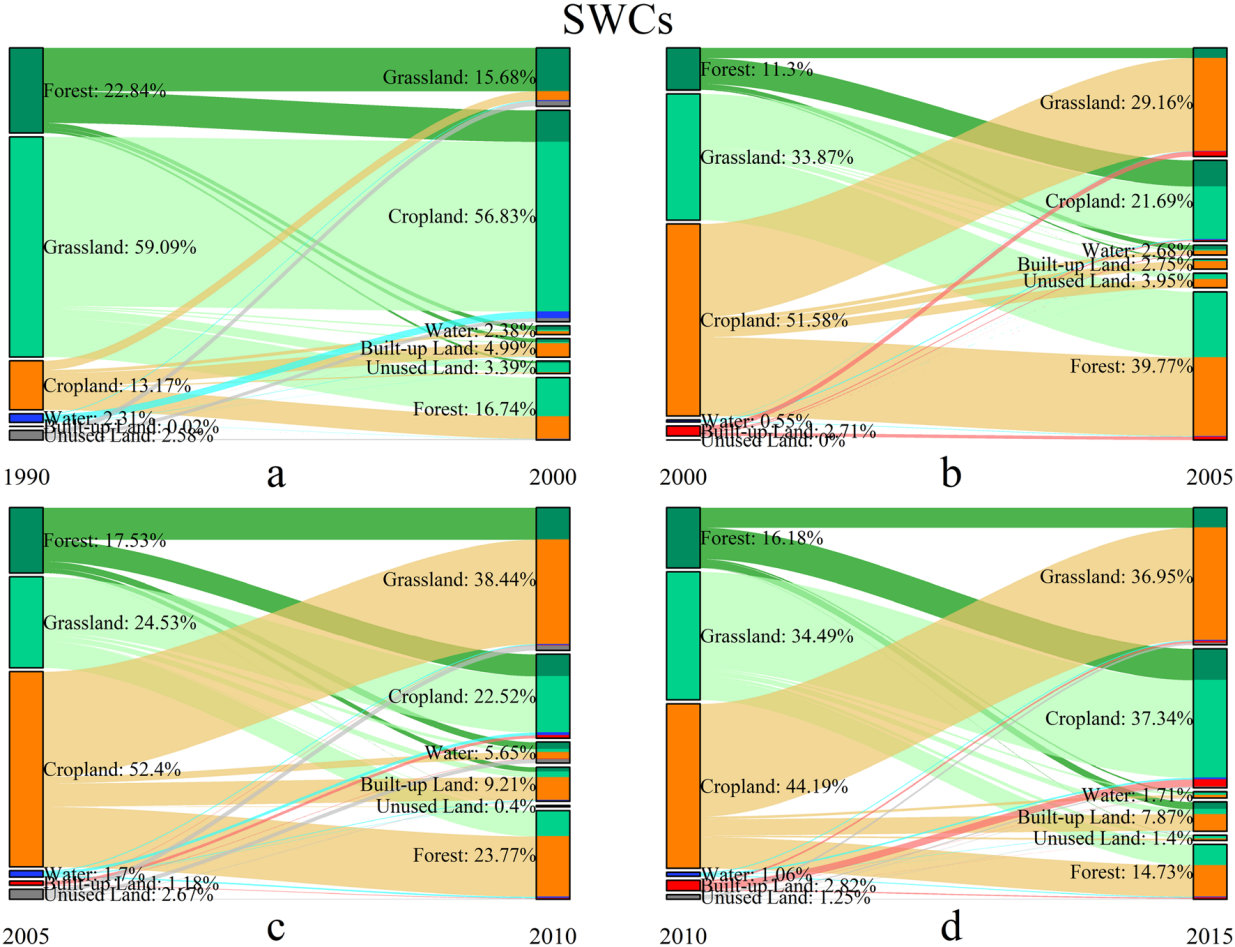
The landscape transformation in SWCs during 1990–2015.

**Figure 8:**
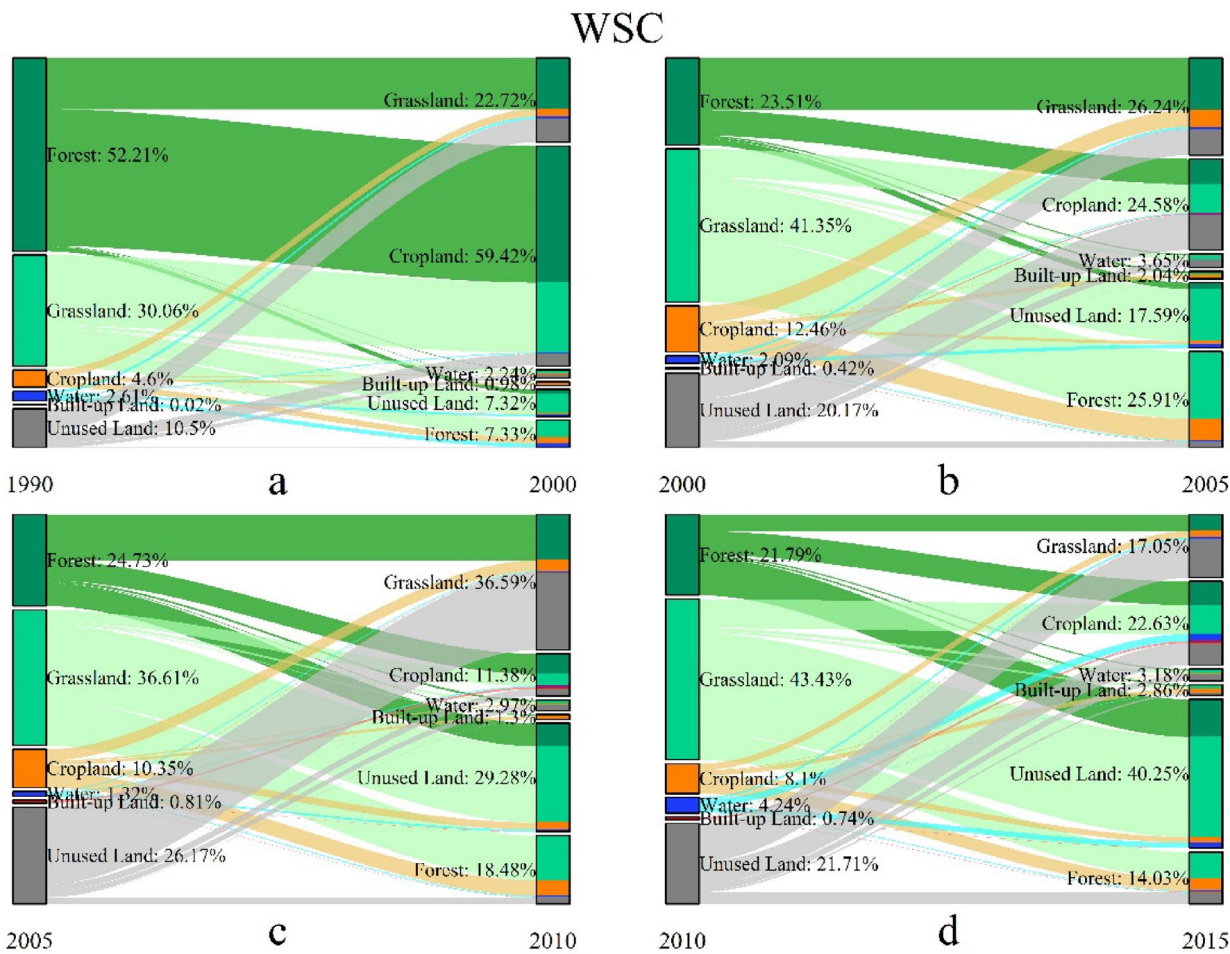
The landscape transformation in WSCs during 1990–2015.

We further acquired the detail transformation information to depict the evolution characteristics of the four ecoregions. The spatial pattern of the landscape conversions in four-type ecoregions during 1990–2015 are shown in Figs. 5, 6, 7 and 8, and the details of the landscape transformations are represented in Tables 6–21 (Appendix). The total landscape conversion area in the four periods is 63,714.02 km^2^, 72,403.09 km^2^, 704,797.60 km^2^ and 104,770.09 km^2^ respectively, showing the trend of rising during T1-3 and decreasing in T4. This is inconsistent with the change of landscape area (Fig. 2), especially in the T2, indicating that the deeper transformations between landscapes is more complicated than their final performance in areas.

To reveal the landscape transformation characteristics of four-type ecoregions, we recorded the landscape transformation process in detail. In WPSFs, grassland and unused land were not only the main turning-out landscape classes, but also the main turning-in landscape during 1990–2015, which are shown in Fig. 5 and Tables 7–10 (Appendix). In the four periods, the area transferred from grassland accounted for about 50% of the total conversion areas, which were 30,149.79 km^2^, 9252.35 km^2^, 217,039.30 km^2^ and 21,541.26 km^2^ respectively. However, the turning-out proportion of grassland continued to decline, while the turning-in proportion was continuously increased. Moreover, the turning-in area compensated for the turning-out area of grassland in T4. In all four periods, the turning-out grassland was basically transformed into cropland and unused land in turn.

The landscape transformations of BMs during 1990–2015 were complex, which are shown in Fig. 6 and Tables 11–14 (Appendix). The total conversion areas were 7885.62 km^2^, 50,084.42 km^2^, 313,461.52 km^2^ and 23,823.32 km^2^ respectively in four periods. During T1, the grassland, forest and unused land were the main turning-out classes. A large amount of forest was transformed to grassland followed by cropland, while grassland and unused land were mainly converted to cropland. In the T2 period, a great deal of mutual transformation had occurred in forest, grassland and cropland. This was the reason why the landscape area seemed not changed (Fig. 4 BMs). Then in the T3, large-scale conversion of grassland was converted to unused land. In the T4 period, the mutual transformation of most landscape classes made the overall dynamic balance except for the increasing turning-in proportion of built-up land. Among the four periods, the turning-out and turning-in area of landscape conversion often achieved dynamic balance, although the landscape conversion was dramatic in BMs. However, during the T3, a large number of grassland was severely degraded into unused land breaking this balance.

In SWCs, the main turning-out and turning-in landscape classes were both cropland, grassland and cropland, which are shown in Fig. 7 and Tables 15–18 (Appendix). The total transformation areas in SWCs were 3067.76 km^2^, 4914.41 km^2^, 9017.70 km^2^ and 10,174.85 km^2^ respectively in four periods. The turning-out of grassland was the most intense followed by forest and cropland in the T1, but the cropland became the most intense one since the T2. The transformation from grassland to cropland occupied the main position of cropland conversion in the T1. The conversion from cropland to grassland had gradually increased and occupied a major position during T2-4. Besides, a large proportion of cropland was suddenly transformed to forest in T2, but then this conversion was gradually reduced. The sudden increase in turning-out of cropland resulted in the enhancement of turning-in of forest in T2-T3. However, the incremental transformation of grassland and forest to cropland compensated the turning-out of cropland in T4.

In WSCs, the total conversion area was 22,610.84 km^2^, 8151.92 km^2^, 165,279.09 km^2^ and 49,230.59 km^2^ respectively in each periods, showing a growth peak in T3, which are shown in Fig. 8 and Tables 19–22 (Appendix). During four periods, the forest, grassland and unused land were the main turning-out classes. The forest was mainly converted to cropland and grassland in T1 and T2, but the conversion from forest to unused land was constantly increased. The grassland was mainly converted to cropland, unused land and forest, especially to the unused land in T3 and T4. The unused land was mainly transformed to grassland and then cropland during four periods. Moreover, the turning-out proportion of forest was gradually decreased during T1-4, and the turning-in proportion was increased first and then decreased. The turning-out proportion of grassland was continuously decreased while turning-in proportion was increased until T3, but the turning-in of grassland cannot always make up for the turning-out. The turning-out and turning-in of unused land were both increased. These trends had led to a continuous increase in total landscape change area from T2 (Fig. 4), especially in the T4, which had less conversion intensity than T3.

### The evolution process of ecological landscape pattern

**Figure 9:**
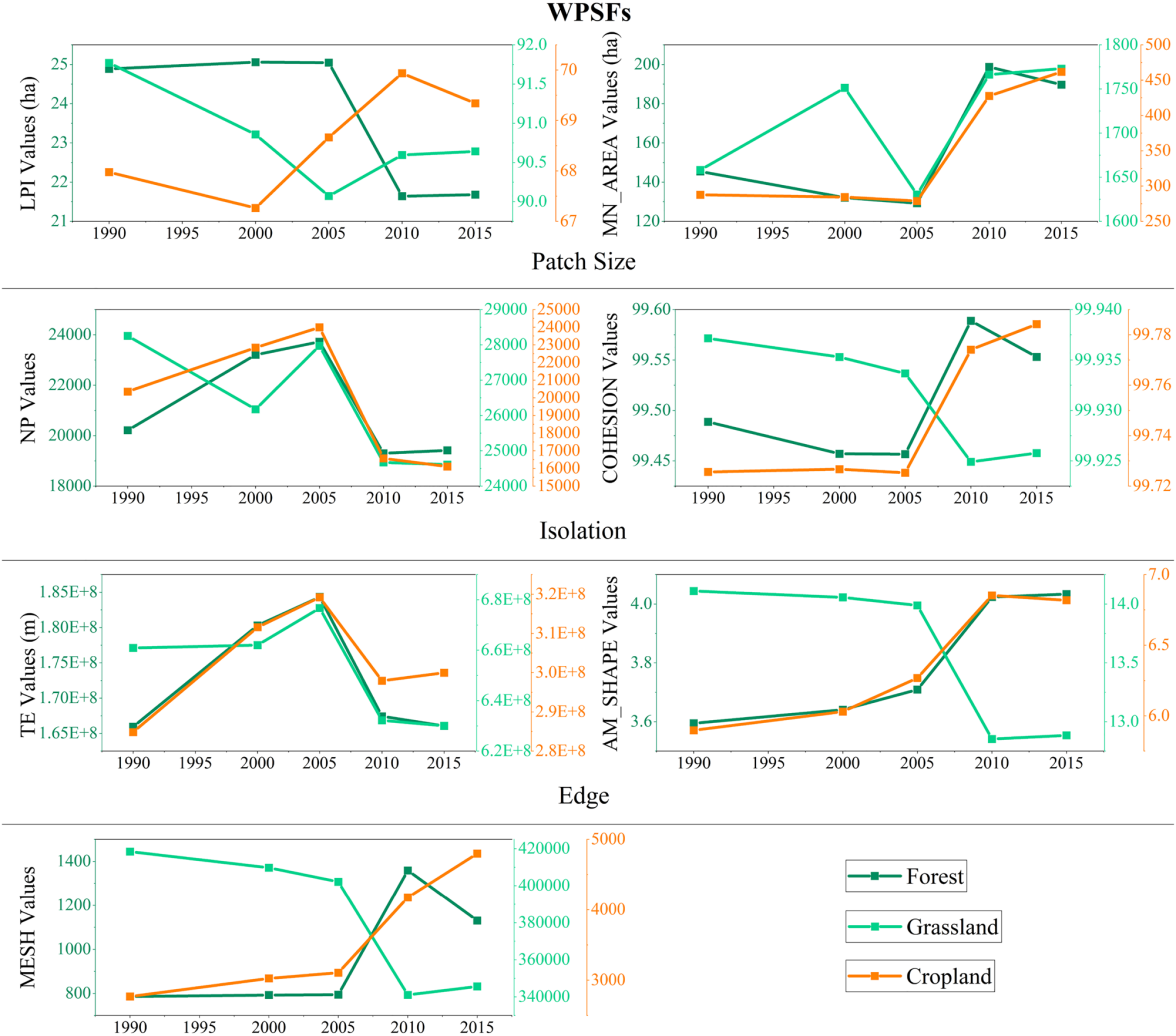
The spatial characteristics of habitats in WPSFs during 1990–2015.

We calculated the selected landscape pattern indices based on section “Methodology” to depict the evolution of landscape pattern in each ecoregions, and the detailed results are shown in Tables 22–25 (Appendix). The habitats patterns of the WPSFs had undergone different evolutions from 1990 to 2015 as shown in Fig. 9. The grassland was the dominant habitat type in WPSFs (Fig. 3). The LPI, NP, COHESIOIN, TE, AM_SHAPE and MESH of grassland decreased to some extent, but the MN_AREA increased. These changes indicate that the grassland was continuously degraded and fragmented: (a) large grassland patches were constantly eroded; (b) the smaller grassland patches gradually were disappeared and cohesion was decreased causing high isolation; (c) the remaining patches boundaries were also eroded as the reduction of edge length. That is the fragmentation of grassland was intensified.

The NP of cropland was decreased, and the other landscape indices values were increased, indicating that the old patches of cropland were expanded outward and the cropland was agglomerated. The LPI of forest were decreased significantly, while the MN_AREA, AM_SHAPE, COHESION and MESH were increased. The NP and TE were increased first and then decreased, which represented that the forest had undergone a process from expansion to agglomeration but the largest forest patch was eroded.

**Figure 10:**
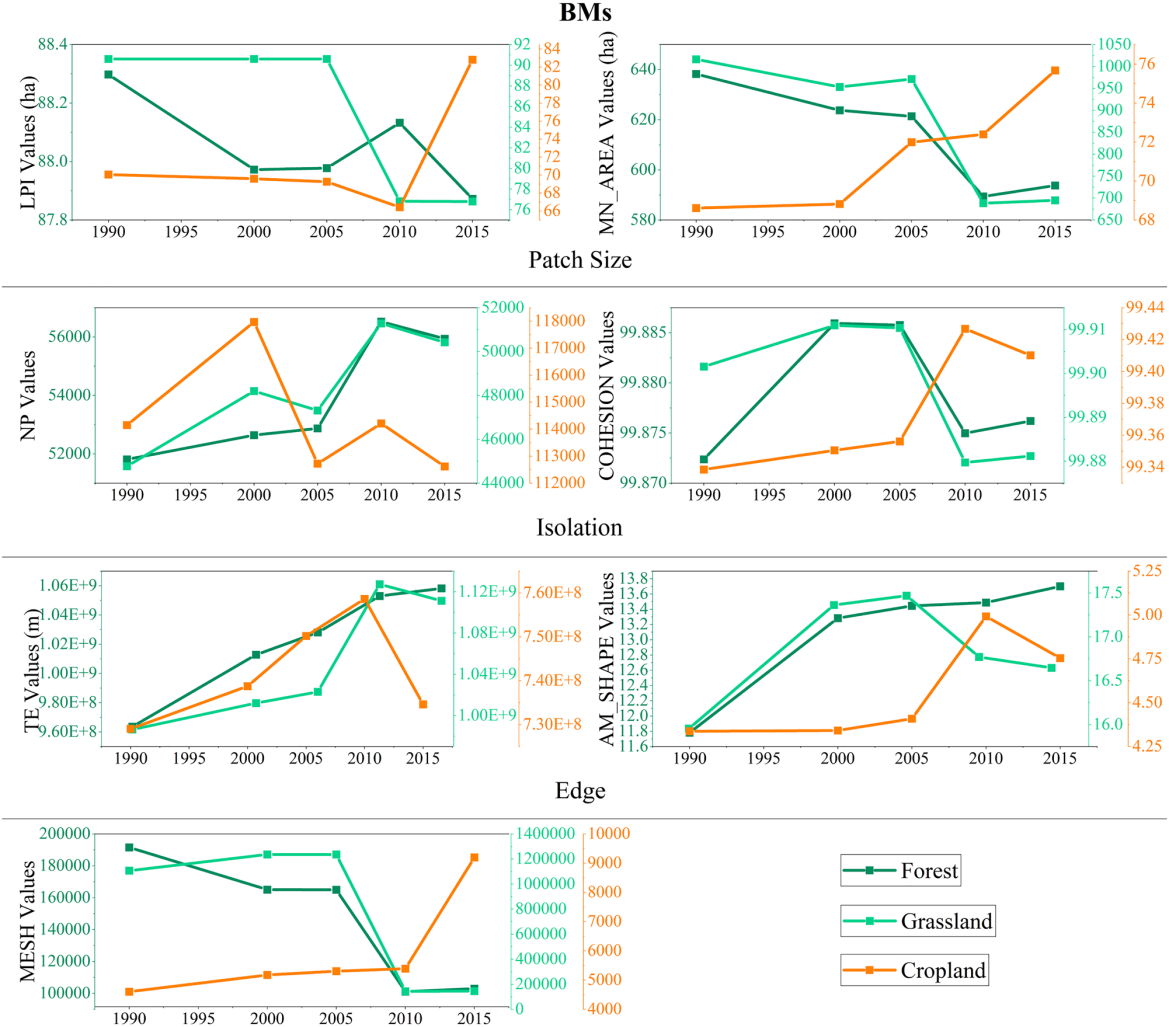
The spatial characteristics of habitats in BMs during 1990–2015.

The pattern characteristics of habitats in BMs during 1990–2015 were shown in Fig. 10. The LPI, MN_AREA, COHESION and MESH of grassland were decreased, but the NP, TE, and AM_SHAPE were increased, indicating that grassland was gradually degenerating and fragmentation as reduction of patch size and increment of isolation and edge. For forest, the LPI, MN_AREA and MESH were decreased, while other landscape indices were almost increased, which indicates that the forest was developed towards fragmentation that mainly reflected in reduction of patch size and increased edge. The LPI, MN_AREA and MESH of cropland were increased obviously, while NP was decreased. That indicates the cropland had undergone consolidation and changed from discrete structure to agglomeration.

**Figure 11:**
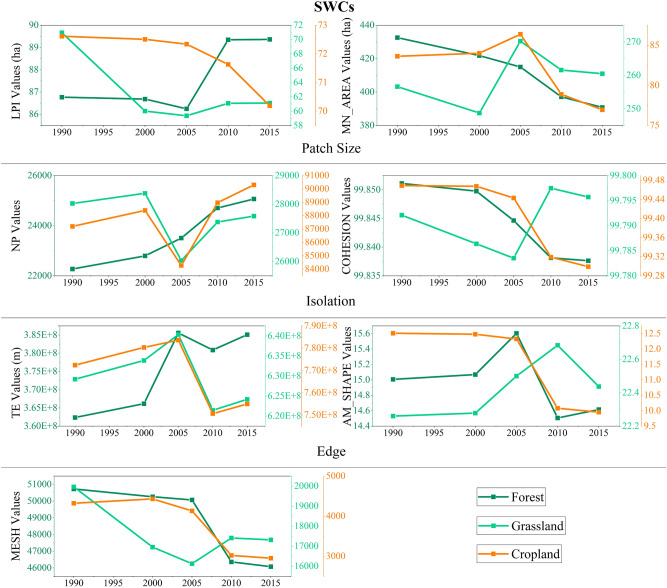
The spatial characteristics of habitats in SWCs during 1990–2015.

In the SWCs, the principal changes of forest were the reduction of MN_AREA, COHESION and MESH, but increment of NP and TE (Fig. 11). The forest had experienced continuous fragmentation. The landscape indices of grassland had almost experienced large fluctuations in T1-3, which the NP, COHESION and MESH decreased first and then increased, while MN_AREA and TE were opposite. This indicates that the grassland was firstly towards fragmented because of the increment of isolation and edge caused by disappearance of small patches, but then the situation changed in the opposite direction in T3. In addition to the significant increase in the NP of cropland, other indices showed a downward trend, indicating that cropland had typical fragmentation characteristics during 1990–2015.

**Figure 12:**
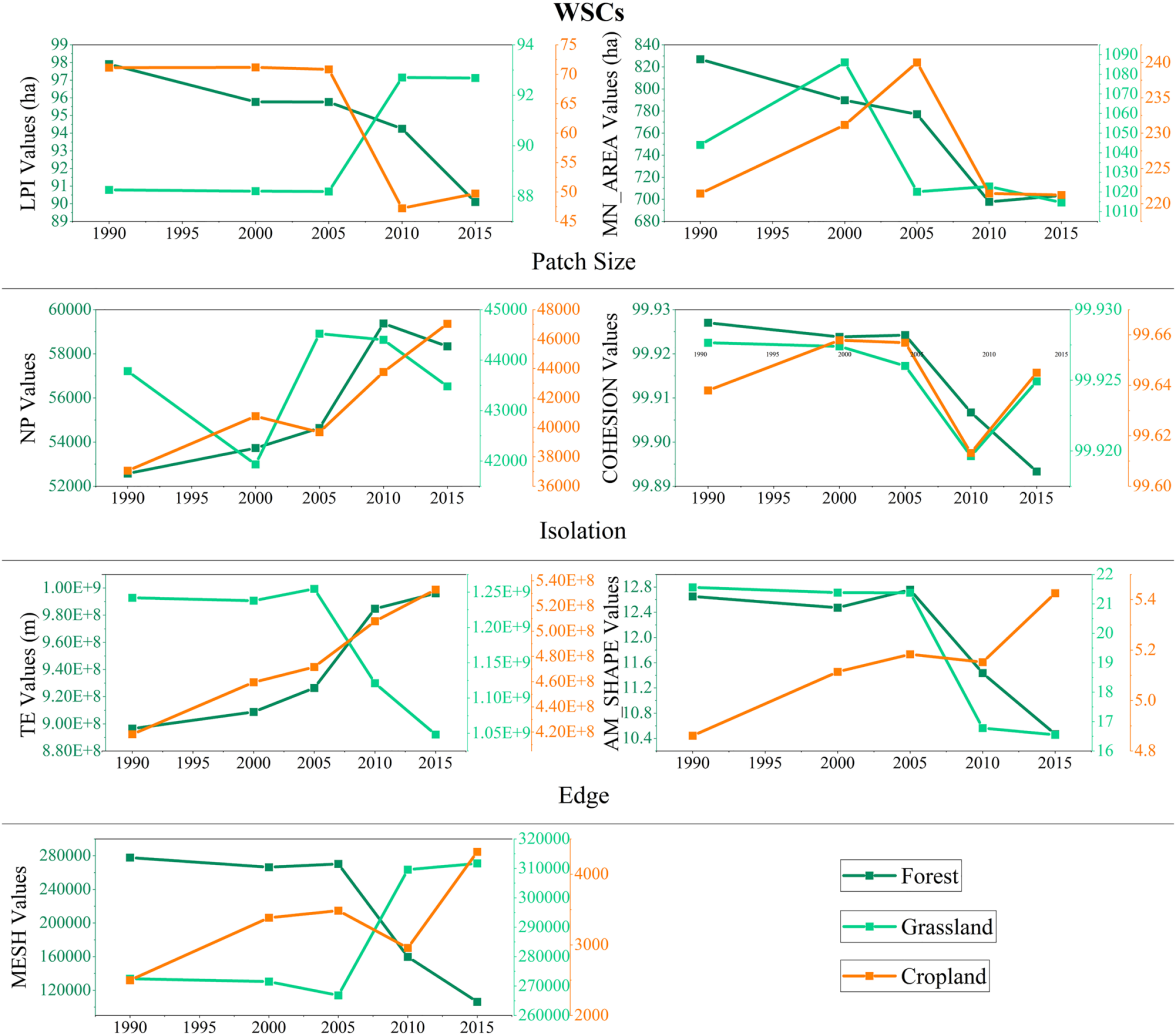
The spatial characteristics of habitats in WSCs during 1990–2015.

In WSCs, except for the increase of the LPI and MESH of grassland, other indices showed a downward trend, especially the TE and AM_SHAPE, indicating that the grassland was developing in the opposite direction of fragmentation mainly caused by increment of patch size and reduction of edge (Fig. 12). Except for the significant increase in the NP and the TE of forest, the remaining indices were declining, indicating that the pattern of forest was significantly more fragmented from reduction of patch size and increment of isolation and edge. As for cropland, the NP, TE and AM_SHAPE were constantly increased, and LPI was significantly decreased, indicating the cropland was also more and more fragmented with landscape expansion.

### Landscape evolution in KEZs during 1990–2015

#### WPSFs: destruction and deterioration of grassland

In the WPSFs, the main habitat type is grassland. The total area of grassland of was almost continuously destroyed, especially in the T3, but had a little increase in T4. Moreover, the pattern of grassland was constantly towards fragmentation. However, in T4, grassland had a slight trend of developing against fragmentation mainly caused by increment of patch size and reduction of isolation compared to T3.

On the contrary, the cropland had undergone a continuous process of agglomeration and expansion according to the evolution of area and pattern during all four periods. The unused land was continuously expansion, especially in T3, but unused land was reduced in T4. The forest had undergone a process from expansion to agglomeration with growth of area mainly caused by the turning-in of grassland and cropland before T4, but the area of forest was reduced because of the increase of turning-out to grassland in T4.

#### BMs: increased fragmentation of habitats

The BMs is mainly composed of grassland and forest. The landscape transformations of BMs during 1990–2015 were complex and easily approaching dynamic balance landscape resulting in landscape area seemed not changed. Nevertheless, substantial landscape area changes happened in the T3: the reduction of the grassland, the increase of unused land, a small increase of water and forest. That is because the large-scale conversion of grassland was converted to unused land.

The main trend of landscape pattern change of grassland was gradually towards fragmentation. This trend was the most intense in T3, but it had been ameliorated in T4 mainly caused by reduction of isolation and edge. Besides, the forest was developing towards fragmentation but this trend had also been alleviated in T4, which mainly caused by reduction of isolation in T4. However, the cropland had undergone consolidation and changed from discrete structure to agglomeration.

#### SWCs: the transformation from cropland to habitats

Most area of the SWCs is occupied by the forest, grassland and cropland. The area of forest had a large increase in the T2 and T3, but a slight decrease in T1 and T4. That is because, on the one hand, a large proportion of cropland was suddenly transformed to forest in T2, but then this conversion was gradually reduced. On the other hand, the turning-out proportion of grassland to forest was suddenly increased in T2. Moreover, the forest had experienced continuous fragmentation.

The grassland area was decreased in T1-2 but increased in T3-4. The pattern evolution of grassland was fragmented in T1-2, but towards opposite direction in T3. Moreover, the area of cropland was declined after the T1, and the pattern was continuously fragmented. Besides, the areas of water and built-up land had continued to increase and improved significantly compared to the initial stage. The growth of built-up land was mainly converted from cropland, and this growth became main landscape area change in T4.

#### WSCs: destruction of habitats

The WSCs is dominated by the grassland, forest and unused land. During 1990–2015, the area changes in WSCs were mainly the growth of cropland and unused land with the reduction of forest and grassland. Besides, the area of water was increased in T2-3, but decreased in T1 and T4.

The grassland was developing to against fragmentation since T4, which mainly caused by reduction of edge. The patterns of forest and cropland were becoming more and more fragmented, but the former was due to destruction, while the latter was due to expansion.

## Discussions

### Is China's ecological environment really developing towards better?

Some recent large-scale studies revealed China is greening, which led in greening of the world. The net change in leaf area of China increased by 17.8% during 2000–2017, and this change accounts for 25% of the global net increase in leaf area^14^. Another research suggests that greening trend existed earlier (1982–2009) in China^17^. This growth of leaf area in China may mainly caused by the increase of forests and croplands. Based on the latest national forest inventory, China’s forest area had increased by about 3.19105 km^2^ during 1977–2008^65^. Tree canopy of China had gained 34% from 1982 to 2016^15^. As for the greening caused by cropland in China, it was mainly determined by the increase of harvest frequency, while the change of cropland area was relatively stable^66^. Therefore, China seemed to be greening intuitively, according to the analysis obtained by direct processing of satellite data.

According to above researches, the most noteworthy question is still difficult to answer: is China's ecological environment really getting better under the background of China becoming green. The above long-time series and large-scale researches focused on the revelation of appearance based on statistical results by some indices, i.e. leaf area index, tree canopy cover, short vegetation, etc., which are direct calculated based on satellite data. The internal transformation mechanisms and detailed spatial characteristics of landscape classes are thus not depicted distinctly, which will lead to one-sided understanding of ecological environment change. Therefore, the long-term and large-scale characterization from a landscape perspective is still lacking, which will lead to a lack of a comprehensive understanding of China’s ecological changes.

In this study, the long-time series and large-scale landscape evolution in KEZs with detailed landscape classification is analyzed to depict more clearly how the ecological environment changed. The KEZs, which are accounting for 40.2% of China’s land area, can be used to reveal whether China's ecological environment has become better. This is because the KEZs are the areas where ecosystems are fragile or have important ecological functions across the country. The previous results show the greening in China mainly occurs in the southeast^14,17^, which is rarely included in the KEZs. These greening areas themselves have the basis of climatic conditions conducive to the ecological environment, which are close to the sea and belong to the monsoon climate. To better answer the question whether China's ecological environment really has became better, it is necessary to reveal the ecological evolution of fragile and important areas, i.e. KEZs.

According to our results, we found a general evolving trend in all four type ecoregions of the KEZs: the ecological environment of most of KEZs had gradually deteriorated until T3 (2005–2010), but this evolution state in WPSFs and BMs were mitigated and even in the opposite direction in T4 (2010–2015). That is, in WPSFs, the main ecological landscape, i.e. grassland, was gradually destruction and deterioration caused by expansions of cropland and unused land. Especially in T3, the grassland was substantially greatly damaged due to the erosion of unused land. Nevertheless, the grassland had an improvement on area and pattern in T4, which was resulted by the reduction of unused land and even forest. In BMs, the landscape area evolution was mostly dominated by dynamic balance except for T3 period, which the grassland was substantially deteriorated to unused land. The fragmentation of grassland and forest was gradually increasing, but both them had been alleviated in T4, while the pattern of cropland was constantly towards agglomeration. In SWCs, the landscape evolution was mostly the transformation from cropland to forest and grassland with the fragmentation of cropland and forest after T1, but this trend became more and more weakening because of the increase of transformation of cropland to built-up land. The areas of water and unused land were constantly increased, but the grassland was always towards fragmentation except in T3. Moreover, the ecological environment in WSCs had been deteriorating, mainly manifested in the continuous conversion of forest and grassland to cropland and unused land. This trend made the grassland more agglomeration since T4, but made the forest more fragmentation.

In summary, the ecological environment evolution of KEZs was towards wore during 1990–2010. Although consistent with the results of some studies, the ecological environment has improved in some areas during 2010–2015, the ecological condition is still not optimistic^67^. Hence, it is still hard to say that China’s ecological environment is developing in a good direction, because the landscape condition of the areas where ecosystems are fragile or have important ecological functions have not improved significantly.

### The effects of KEZs plan

The KEZs plan, which is implemented in 2010, summarizes and identifies the ecoregions based on their historical ecological characteristics. From the perspectives of landscape pattern, the ecological environment in KEZs was increasingly unfavorable in 1990–2010, but most of the areas had been improved to varying degrees in 2010–2015 after the implementation of KEZs plan. This result seems to be an evidence to confirm the positive impact of the human subjective activities, i.e. policy plan.

The KEZs plan is a strategic, basic and restrictive plan for China's territory development plan. It is a powerful policy that is compiled by the State Council of China and implemented by the cooperation of various departments, which represents the determination of the Chinese government to protect the ecological environment. Some of the specific measurements have been taken to ensure the implementation. For example, the government implements ecological transfer payment, which is mainly used to strengthen the production capacity of ecological products in KEZs, especially the central and western KEZs. The central government had arranged a total of 251.3 billion yuan in transfer payments from 2008 to 2015^68^. Secondly, under the guidance of the plan, the competent authority shall strictly examine and approve, control and supervise the use of land. Specifically, it is manifested in limiting the spatial scope of construction land development, gradually reducing the space occupied by rural residential areas, and freeing up more space for maintaining the virtuous cycle of the ecosystem, and so on, Moreover, the government implements an active population withdrawal policy to guide a part of the population to transfer to urbanized areas. While improving the lives of local residents, the interference of human activities is restricted to the greatest extent. Meanwhile, some existing ecological protected policies are coupled in KEZs, i.e. the natural forest conservation program (NFCP), the grain to green program (GTGP), returning farmland to lakes and other measures. These measures have been proven to have positive ecological and social significance^20,21^. Besides, there are also more measures have been taken for the implementation of KEZs^25^.

**Figure 13:**
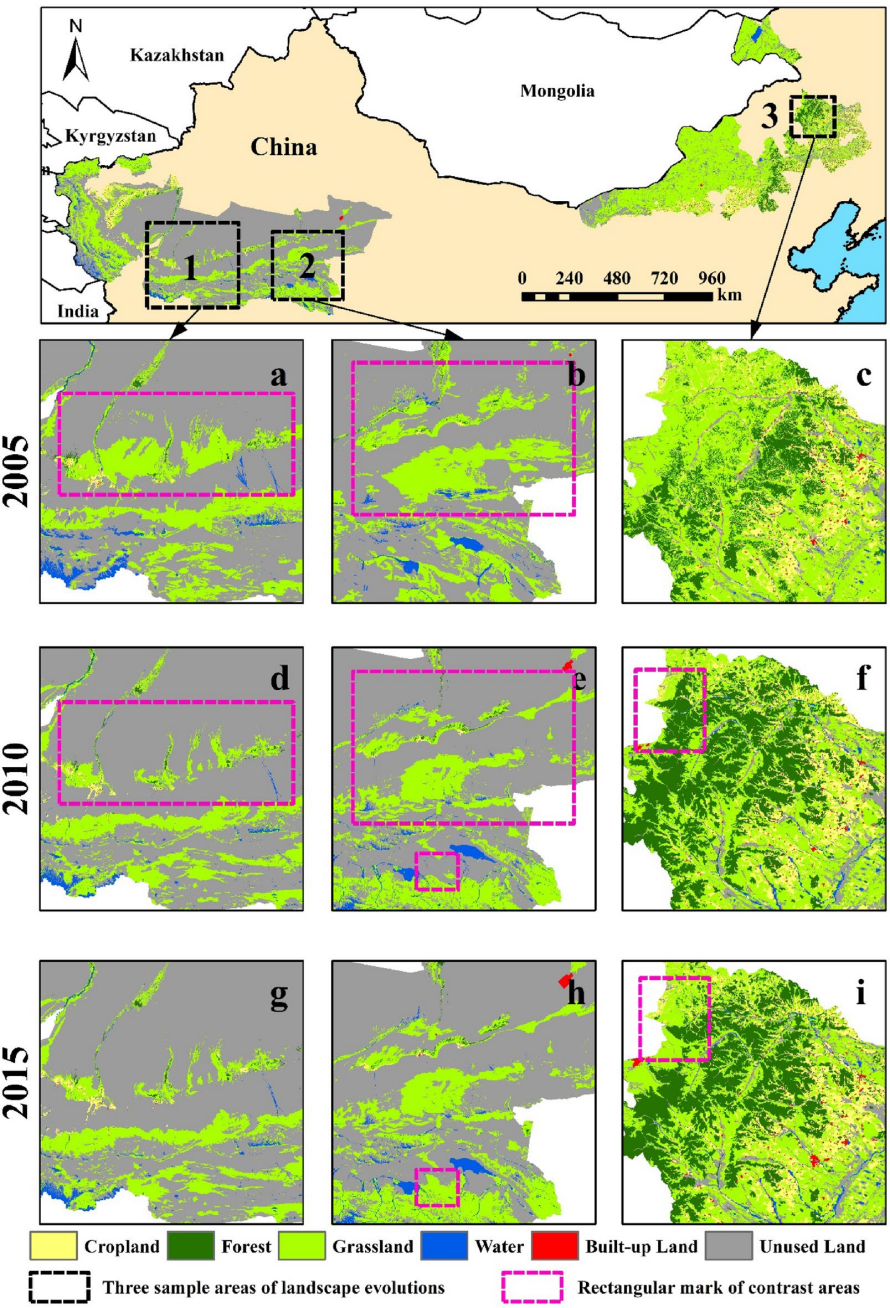
Three sample areas in WPSFs in 2005, 2010 and 2015 for exploring the impact of KEZs plan on landscape evolution. (**a**–**c**) The sample areas in 2005, (**d–f**) in 2010 and (**g**–**i**) in2015. The areas where the landscape evolution were more prominent between the two points is signed by rectangular marks. The map was created in ArcMap 10.7 (www.arcgis.com).

For detailed depicting the influence of this policy, we thus compare the landscape evolution characteristics between before and after the implementation of the KEZs. In all KEZs, the grassland was greatly degraded during the T3 period, but this degradation was curbed during after the implementation of KEZs plan (Fig. 2b). We also find the landscape evolution characteristics in WPSFs are obviously differentiated between T3 (2005–2010) and T4 (2010–2015), and three prominent areas are as shown in Fig. 13. In T3, grassland was seriously and generally eroded by unused land with reduction of area and fragmentation (Fig. 13a and d). This general trend remained unchanged, although some areas had a little growth and connection of grassland (Fig. 13b and e). The water area had also been reduced to some extent, but forest had been increased transformed from grassland (Fig. 13c and f). It should be noted that this was basically the common evolution trend of grassland in T1-3. However, this trend of grassland had been stopped (Fig. 13d and g), and even developed in the opposite direction, i.e. the net increase and concentration (Fig. 13e and h) after the implementation of the KEZs plan. It is a pity that some forests are degraded into grasslands (Fig. 13f and i). Therefore, the long-term grassland evolution trend had been changed, and the area and pattern of the main habitat landscape grassland had been improved. This is a huge ecological benefit for the WPSFs, which is dominated by unused land with a weak ecological landscape basis. Besides, in BMs, the continuous fragmentation of habitats (T1-3) and the large-scale deterioration of grassland to unused land (T3) were inhibited after the implementation of the KEZs plan.

However, the improved effect of this plan is not obvious in SWCs and WSCs during 2010–2015. The landscape evolution had not changed towards an improved favorable direction to enhance ecological function during T4 (sections “SWCs: the transformation from cropland to habitats” and “WSCs: destruction of habitats”). Therefore, due to the fragile ecological landscape basis of WPSFs and the severely deterioration of grassland of BMs during in T3 (Fig. 3), we assumed that the policy is more prominent in prohibiting sabotage and protecting areas with fragile ecological bases. This may also be related to the fact that more transfer payments have been received in these areas, because some or most of WPSFs and BMs are mainly located in the central and western part of China.

For promoting better evolutions in future KEZs, some measures, i.e., stricter land use control, regional balance of ecological transfer payments and deepening of ecological migration policies, etc., need to be implemented and deepened. In the WPSFs and the BMs, the erosion of farmland to grassland should be controlled on the basis of current evolution, especially in avoiding grassland fragmentation. In SWCs, the growth of built-up land should be constrained coordinating with the effective implementation of the programs of returning farmland to forest and grassland. In WSCs, Agricultural production activities at the cost of sacrificing habitats should be restricted, through the inclining ecological transfer payment to areas with less access before, i.e. WSCs, and deepening of ecological migration activities.

There are also some limitations in this study due to the available data. We only obtained the interpretation data of remote sensing satellites before 2015, so that our analysis of ecological environment focused on the depicting the ecological evolution in KEZs from a long-time and large-scale landscape perspective that we considered it is important and ignored by existing researches. Then the other ecological characteristics based on other data were not taken into account. The KEZs in this refers specifically to the key ecological function zones in limited development zones, not prohibited development zones where the zones are also including some key ecological function. This is because the key ecological function zones in prohibited development zones already has a good ecological foundation and were listed as national protection areas to protect at an earlier time. Furthermore, some counties have also been newly added to key ecological function areas in 2016, but we did not analyze these regions because the data limitations.

## Conclusions

For comprehensively exploring the environmental changes in China, especially in KEZs, it is desirable to depict the entire landscape pattern evolution. The conclusions and suggestions of landscape evolution in KEZs during 1990–2015 are obtained as follows: (a) In WPSFs, the landscape evolution of WPSFs was mainly the destruction and deterioration of grassland caused by expansions of cropland and unused land. (b) In BMs, the grassland was substantially deteriorated to unused land especially in T3, and the fragmentation of grassland and forest was gradually increasing. (c) In SWCs, the landscape evolution was mostly the transformation from cropland to forest and grassland with the fragmentation of cropland and forest after T1. (d) In WSCs, the main landscape evolution was mostly the transformation from forest and grassland to cropland and unused land. Around the extensive invasion of grassland and forest, the occupation of grassland and forest by cropland should be stopped. (e) The entire KEZs ecological environment had gradually deteriorated, but most of the areas had been improved to varying degrees in 2010–2015 after the implementation of KEZs plan.

Even if some existing researches proved China is greening, it is still hard to say that China’s ecological environment is developing in a good direction, because the landscape condition of the areas where ecosystems are fragile or have important ecological functions have not improved significantly. The government has adopted many measures to implement the plan, such as ecological transfer payment, land use control, population migration, coupling other environmental protection policies, etc. We found the impact of KEZs is more inclined to protect ecologically fragile areas than to improve the ecological environment of the original high-quality ecological service areas. This may be due to the fact that the more transfer payments have been received in the ecoregions in central and western part of China where the ecological environment is fragile. We also made some planning suggestions, such as stricter land use control, regional balance of ecological transfer payments and deepening of ecological migration policies, etc., were proposed for promoting better future environment changes according to local ecological evolution characteristics. Future research will conduct long-term and more comprehensive of ecological analysis in KEZs for depicting the continuous impact of this plan. The impact of this plan on the global scale will also be taken into account to analyze.

### Supplementary Information

Supplementary Information.

## Data Availability

The land use maps of China that support this study are available for a fee from Resource and Environment Science and Data Center (https://www.resdc.cn/DOI/DOI.aspx?DOIID=54).

## References

[1] Andren H (1994). Effects of habitat fragmentation on birds and mammals in landscapes with different proportions of suitable habitat: A review. Oikos.

[2] Kriska T, Levchenko VV, Chu FF, Esworthy RS, Girotti AW (2008). Increasing human dominance of tropical forests. Free Radic. Biol. Med..

[3] Bregman TP, Sekercioglu CH, Tobias JA (2014). Global patterns and predictors of bird species responses to forest fragmentation: Implications for ecosystem function and conservation. Biol. Cons..

[4] Haddad NM (2015). Habitat fragmentation and its lasting impact on Earth’s ecosystems. Sci. Adv..

[5] Yousefi M, Barghjelveh S, Darvishi A, Mobargaee Dinan N (2021). Ecological Sustainability in “Energy Return on Investment (EROI)” and its Correlation with Agricultural Landscape Heterogeneity (Case Study: Qazvin Province). J. Aroecol..

[6] Darvishi A, Yousefi M, Dinan NM, Angelstam P (2022). Assessing levels, trade-offs and synergies of landscape services in the Iranian province of Qazvin: Towards sustainable landscapes. Landsc. Ecol..

[7] Fan X, Gu X, Yu H (2021). The spatial and temporal evolution and drivers of habitat quality in the Hung River Valley. Land.

[8] Foley JA (2005). Global consequences of land use. Science.

[9] Kareiva P, Watts S, McDonald R, Boucher T (2007). Domesticated nature: Shaping landscapes and ecosystems for human welfare. Science.

[10] Ostrom E (2009). A general framework for analyzing sustainability of social-ecological systems. Science.

[11] Ceballos G (2015). Accelerated modern human-induced species losses: Entering the sixth mass extinction. Sci. Adv..

[12] Darvishi A, Yousefi M, Mobarghei-Dinan N (2020). Investigating the effect of Socio-economic disturbance resulting from human activities on landscape ecological function using HANPP index (Case Study: Qazvin Province). J. Nat. Env..

[13] Yousefi M (2021). Comparison of two biophysical indicators under different landscape complexity. Ecol. Indic..

[14] Chen C (2019). China and India lead in greening of the world through land-use management. Nat. Sustain..

[15] Song XP (2018). Global land change from 1982 to 2016. Nature.

[16] Zhang Y (2016). Multiple afforestation programs accelerate the greenness in the ‘Three North’region of China from 1982 to 2013. Ecol. Indic..

[17] Piao S (2015). Detection and attribution of vegetation greening trend in China over the last 30 years. Glob. Change Biol..

[18] Fu B, Chen L, Ma K, Zhou H, Wang J (2000). The relationships between land use and soil conditions in the hilly area of the loess plateau in northern Shaanxi, Chins. Catena.

[19] Pauly D (2003). The future for fisheries. Science.

[20] Jianguo L, Jared D (2005). China’s environment in a globalizing world. Nature.

[21] Liu J, Li S, Ouyang Z, Tam C, Chen X (2007). Ecological and socioeconomic effects of China’s policies for ecosystem services. Proc. Natl. Acad. Sci..

[22] Wu X, Liu H (2013). Consistent shifts in spring vegetation green-up date across temperate biomes in China, 1982–2006. Glob. Change Biol..

[23] Liu D, Chen Y, Cai W (2014). The contribution of China’s Grain to Green Program to carbon sequestration. Landsc. Ecol..

[24] Jia X (2014). The tradeoff and synergy between ecosystem services in the Grain-for-Green areas in Northern Shaanxi, China. Ecol. Indic. J..

[25] The State Council of the People's Republic of China. Notice of the State Council on Printing and Distributing the National Main Functional Area Plan (46). https://www.gov.cn/zwgk/2011-06/08/content_1879180.htm (2010).

[26] Zhang J (2023). Analysis of spatio-temporal pattern changes and driving forces of Xinjiang plain oases based on geodetector. Land.

[27] Ries RUTHDEF, Ansen ANH, Urner BLT, Eid ROR (2007). Land use change around protected areas: Management to balance human needs and ecological function. Ecol. Appl..

[28] Umming GRSC, Llen CRRA (2017). Protected areas as social-ecological systems: Perspectives from resilience and social-ecological systems theory. Ecol. Appl..

[29] Curran LM, Trigg SN, Mcdonald AK, Astiani D (2004). Lowland forest loss in protected areas of Indonesian Borneo. Science.

[30] Loveland TR, Merchant JM (2004). Ecoregions and ecoregionalization: Geographical and ecological perspectives. Environ. Manage..

[31] Hansen AJ, Defries R (2007). Ecological mechanisms linking protected areas to surrounding lands. Ecol. Appl..

[32] Ban NC (2017). Social and ecological effectiveness of large marine protected areas. Glob. Environ. Change.

[33] Omernik JM, Bailey RG (1998). Distinguishing between watersheds and ecoregions ’ holistic approaches to research, assess, monitor, misuse and misunderstanding of watersheds for structuring ecological research and management tant purposes and are complementary when used ecoregions r. J. Am. Water Resourc. Assoc..

[34] Obbink RB (2010). Global assessment of nitrogen deposition effects on terrestrial plant diversity: A synthesis. Ecol. Appl..

[35] Fischer J, Lindenmayer DB (2007). Landscape modification and habitat fragmentation: A synthesis Joern Fischer* and David B. Lindenmayer Centre. Glob. Ecol. Biogeogr..

[36] Tscharntke T (2012). Landscape moderation of biodiversity patterns and processes—eight hypotheses. Biol. Rev..

[37] Ibáñez I, Katz DSW, Peltier D, Wolf SM, Connor-Barrie BT (2014). Assessing the integrated effects of landscape fragmentation on plants and plant communities: The challenge of multiprocess-multiresponse dynamics. J. Ecol..

[38] Yousefi M (2020). An energy-landscape integrated analysis to evaluate agroecological scarcity. Sci. Total Env..

[39] Darvishi A, Mobarghaee-Dinan N, Barghjelveh S, Yousefi M (2020). Assessment and spatial planning of landscape ecological connectivity for biodiversity management (Case study: Qazvin province). Iran. J. Appl. Ecol..

[40] Ries L, Fletcher RJ, Battin J, Sisk TD (2004). Ecological responses to habitat edges: Mechanisms, models, and variability explained. Annu. Rev. Ecol. Evol. Syst..

[41] Sih A, Ferrari MCO, Harris DJ (2011). Evolution and behavioural responses to human-induced rapid environmental change. Evol. Appl..

[42] Mitchell MGE (2015). Reframing landscape fragmentation’s effects on ecosystem services. Trends Ecol. Evol..

[43] Saura S, Pascual-Hortal L (2007). A new habitat availability index to integrate connectivity in landscape conservation planning: Comparison with existing indices and application to a case study. Landsc. Urban Plan..

[44] Fahrig L (1985). Effects of habitat fragmentation on biodiversity. Rev. Chil. Histor. Nat..

[45] Fahrig L (2017). Ecological responses to habitat fragmentation per se. Annu. Rev. Ecol. Evol. Syst..

[46] Darvishi A, Mobarghaee N, Yousefi M, Barghjelveh S (2021). Using the method of “Effective Mesh Size” for qualitative evaluation of regional protected areas (Case study: Qazvin province). J. Environ. Stud..

[47] Imbernon J, Branthomme A (2001). Characterization of landscape patterns of deforestation in tropical rain forests. Int. J. Rem. Sens..

[48] McGarigal, K. & Marks, B. J. FRAGSTATS: Spatial pattern analysis program for quantifying landscape structure. In *General Technical Report—US Department of Agriculture, Forest Service* **97331**, 67 (1995).

[49] Zhang S, Zhang J, Li F, Cropp R (2006). Vector analysis theory on landscape pattern (VATLP). Ecol. Model..

[50] Darvishi A, Yousefi M, Mobarghaee-Dinan N (2021). Evaluating the correlation between pollination ecosystem service and landscape pattern metrics (Case study: Qazvin province). Iran. J. Appl. Ecol..

[51] Krummel JR, Gardner RH, Sugihara G, Oneill RV, Coleman PR (1987). Landscape patterns in a disturbed environment. Oikos.

[52] O’Neill RV (1988). Indices of landscape pattern. Landsc. Ecol..

[53] Plotnick RE, Gardner RH, O’Neill RV (1993). Lacunarity indices as measures of landscape texture. Landsc. Ecol..

[54] Matsushita B, Xu M, Fukushima T (2006). Characterizing the changes in landscape structure in the Lake Kasumigaura Basin, Japan using a high-quality GIS dataset. Landsc. Urban Plan..

[55] Riitters KH (1995). A factor analysis of landscape pattern and structure metrics. Landsc. Ecol..

[56] Xu, X. *et al.* China multi-period land use remote sensing monitoring dataset (CNLUCC). In *Resource and Environmental Science Data Registration and Publication System (*http://www.resdc.cn/DOI*)* (2018).

[57] Leita ÂB, Ahern J (2002). Applying landscape ecological concepts and metrics in sustainable landscape planning. Landsc. Urban Plan..

[58] Wang X, Blanchet FG, Koper N (2014). Measuring habitat fragmentation: An evaluation of landscape pattern metrics. Methods Ecol. Evol..

[59] Tischendorf L (2001). Can landscape indices predict ecological processes consistently?. Landsc. Ecol..

[60] Jaeger JAG (2000). Landscape division, splitting index, and effective mesh size: New measures of landscape fragmentation. Landsc. Ecol..

[61] Li X (2005). The adequacy of different landscape metrics for various landscape patterns. Pattern Recogn..

[62] Li X (2005). Relationship between landscape structure metrics and wetland nutrient retention function: A case study of Liaohe Delta, China. Ecol. Indic..

[63] Olsen LM, Dale VH, Foster T (2007). Landscape patterns as indicators of ecological change at Fort Benning, Georgia, USA. Landsc.. Urban Plan..

[64] Olsen LM, Washington-Allen RA, Dale VH (2005). Time-series analysis of land cover using landscape metrics. GI Sci. Rem. Sens..

[65] Guo ZD, Hu HF, Li P, Li NY, Fang JY (2013). Spatio-temporal changes in biomass carbon sinks in China’s forests from 1977 to 2008. Sci. China Life Sci..

[66] Ray DK, Foley JA (2013). Increasing global crop harvest frequency: Recent trends and future directions. Environ. Res. Lett..

[67] Busch J (2021). A global review of ecological fiscal transfers. Nat. Sustain..

[68] Ministry of Finance of the People’s Repubic of China. http://www.mof.gov.cn/zhengwuxinxi/caijingshidian/renminwang/201605/t20160519_1996793.htm (2016).

