# Supplementary material for: Landscape evolution in China’s key ecological function zones during 1990–2015
Source: Sci Rep. 2024 Feb 1;14:2655. doi: 10.1038/s41598-024-52863-1 (PMC10834530; doi:10.1038/s41598-024-52863-1)
Supplement: Supplementary file 1 — Supplementary Information. [file 41598_2024_52863_MOESM1_ESM.docx]

# Appendix

Table 1. The five-time class area (CA) of each landscape of total China’s key ecological function zones with the hectare of area unit.

| CA(km^2^) | 1990 | 2000 | 2005 | 2010 | 2015 |
| --- | --- | --- | --- | --- | --- |
| Built-up Land | 12410.7003 | 12841.5303 | 13249.0161 | 15666.1443 | 18683.4285 |
| Cropland | 290133.599 | 314637.992 | 316071.869 | 320635.35 | 334013.612 |
| Forest | 890007.061 | 879539.957 | 881369.583 | 886284.845 | 880085.772 |
| Grassland | 1606839.93 | 1591910.88 | 1588128.21 | 1380073.75 | 1366364 |
| Unused Land | 913845.661 | 913367.875 | 913364.569 | 1093453.62 | 1096916.44 |
| Water | 92048.8887 | 92948.0409 | 93077.4501 | 109845.828 | 109951.615 |

Table 2. The five-time class area (CA) of each landscape of WPSF with the hectare of area unit.

| CA(km^2^) | 1990 | 2000 | 2005 | 2010 | 2015 |
| --- | --- | --- | --- | --- | --- |
| Built-up Land | 4655.0646 | 4643.7057 | 4720.14 | 5201.3547 | 6116.0508 |
| Cropland | 58521.6576 | 64955.0088 | 66910.4685 | 70840.1151 | 74387.4624 |
| Forest | 29398.3731 | 30646.4805 | 30666.4092 | 38365.0254 | 36843.2982 |
| Grassland | 468531.819 | 458442.793 | 456112.956 | 435736.985 | 436334.234 |
| Unused Land | 544775.993 | 546316.458 | 546700.631 | 560884.266 | 557239.865 |
| Water | 31635.2385 | 32513.8644 | 32408.8344 | 26586.684 | 26696.8134 |

Table 3. The five-time class area (CA) of each landscape of BM with the hectare of area unit.

| CA(km^2^) | 1990 | 2000 | 2005 | 2010 | 2015 |
| --- | --- | --- | --- | --- | --- |
| Built-up Land | 1585.2744 | 1660.0518 | 1747.197 | 2157.5952 | 2701.9917 |
| Cropland | 76631.8068 | 81194.4855 | 81152.7084 | 82687.6818 | 86062.968 |
| Forest | 329484.294 | 328331.129 | 328543.451 | 335512.081 | 334800.386 |
| Grassland | 609293.521 | 607444.923 | 607452.864 | 418552.49 | 416982.418 |
| Unused Land | 94162.1553 | 92611.2645 | 92347.9119 | 253314.34 | 251272.396 |
| Water | 31169.6397 | 31044.0285 | 31045.8681 | 50640.7923 | 51088.5693 |

Table 4. The five-time class area (CA) of each landscape of SWC with the hectare of area unit.

| CA(km^2^) | 1990 | 2000 | 2005 | 2010 | 2015 |
| --- | --- | --- | --- | --- | --- |
| Built-up Land | 1442.8449 | 1595.2527 | 1707.6951 | 2432.3391 | 2945.9097 |
| Cropland | 72928.224 | 74267.7525 | 72799.3989 | 70195.7853 | 69498.6012 |
| Forest | 96357.5136 | 96170.3973 | 97570.4805 | 98134.1334 | 97985.7072 |
| Grassland | 71929.7028 | 70598.124 | 70367.5557 | 71622.3681 | 71872.371 |
| Unused Land | 1400.2317 | 1425.195 | 1508.8167 | 1304.1675 | 1319.7861 |
| Water | 1994.2983 | 1996.2729 | 2101.2417 | 2367.4392 | 2433.87 |

Table 5. The five-time class area (CA) of each landscape of WSC with the hectare of area unit.

| CA(km^2^) | 1990 | 2000 | 2005 | 2010 | 2015 |
| --- | --- | --- | --- | --- | --- |
| Built-up Land | 4727.5164 | 4942.5201 | 5073.984 | 5874.8553 | 6919.4763 |
| Cropland | 82051.911 | 94220.7453 | 95209.2936 | 96911.7678 | 104064.581 |
| Forest | 434766.88 | 424391.95 | 424589.243 | 414273.605 | 41045638.1 |
| Grassland | 457084.885 | 455425.045 | 454194.839 | 454161.91 | 441174.973 |
| Unused Land | 273507.281 | 273014.957 | 272807.21 | 277950.851 | 287084.39 |
| Water | 27249.7122 | 27393.8751 | 27521.5059 | 30250.9125 | 29732.3622 |

Table 6. The conversion of landscape classes in WPSF during 1990-2000 with pixel of unit (30 m*30 m).

|  | Forest | Grassland | Cropland | Water | Built-up Land | Unused Land | Unchanged | Total  Turning-out |
| --- | --- | --- | --- | --- | --- | --- | --- | --- |
| Forest |  | 191593 | 488115 | 16182 | 1830 | 133500 |  | 831220 |
| Grassland | 1266079 |  | 8218298 | 1314255 | 61386 | 7160718 |  | 18020736 |
| Cropland | 632933 | 1956125 |  | 50784 | 208657 | 281003 |  | 3129502 |
| Water | 56953 | 780305 | 153154 |  | 794 | 2173635 |  | 3164841 |
| Built-up Land | 3310 | 22447 | 260741 | 861 |  | 14562 |  | 301921 |
| Unused Land | 258747 | 3860003 | 1157248 | 2758927 | 16626 |  |  | 8051551 |
| Unchanged |  |  |  |  |  |  | 1230408678 | 1230408678 |
| Total  Turning-in | 2218022 | 6810473 | 10277556 | 4141009 | 289293 | 9763418 | 1230408678 | 1263908449 |

Table 7. The conversion of landscape classes in WPSF during 2000-2005 with pixel of unit (30 m*30 m).

|  | Forest | Grassland | Cropland | Water | Built-up Land | Unused Land | Unchanged | Total  Turning-out |
| --- | --- | --- | --- | --- | --- | --- | --- | --- |
| Forest |  | 242744 | 268045 | 8072 | 4918 | 73697 |  | 597476 |
| Grassland | 400983 |  | 2451811 | 203558 | 45782 | 2193059 |  | 5295193 |
| Cropland | 148875 | 917100 |  | 12355 | 67334 | 82330 |  | 1227994 |
| Water | 16654 | 160133 | 101450 |  | 13532 | 438779 |  | 730548 |
| Built-up Land | 2518 | 25763 | 28540 | 753 |  | 5224 |  | 62798 |
| Unused Land | 50423 | 1360183 | 550715 | 388902 | 16152 |  |  | 2366375 |
| Unchanged |  |  |  |  |  |  | 1253627334 | 1253627334 |
| Total  Turning-in | 619453 | 2705923 | 3400561 | 613640 | 147718 | 2793089 | 1253627334 | 1263907718 |

Table 8. The conversion of landscape classes in WPSF during 2005-2010 with pixel of unit (30 m*30 m).

|  | Forest | Grassland | Cropland | Water | Built-up Land | Unused Land | Unchanged | Total  Turning-out |
| --- | --- | --- | --- | --- | --- | --- | --- | --- |
| Forest |  | 6395788 | 1585680 | 178046 | 75308 | 1864684 |  | 10099506 |
| Grassland | 14838449 |  | 11129277 | 3478703 | 722833 | 86765955 |  | 116935217 |
| Cropland | 2452037 | 6658908 |  | 406123 | 1139613 | 1473498 |  | 12130179 |
| Water | 193177 | 4329686 | 474755 |  | 23617 | 10267589 |  | 15288824 |
| Built-up Land | 178071 | 458156 | 1013807 | 23244 |  | 170880 |  | 1844158 |
| Unused Land | 991665 | 76427739 | 2292799 | 4727225 | 417463 |  |  | 84856891 |
| Unchanged |  |  |  |  |  |  | 1022755712 | 1022755712 |
| Total  Turning-in | 18653399 | 94270277 | 16496318 | 8813341 | 2378834 | 100542606 | 1022755712 | 1263910487 |

Table 9. The conversion of landscape classes in WPSF during 2010-2015 with pixel of unit (30 m*30 m).

|  | Forest | Grassland | Cropland | Water | Built-up Land | Unused Land | Unchanged | Total  Turning-out |
| --- | --- | --- | --- | --- | --- | --- | --- | --- |
| Forest |  | 2416332 | 634159 | 23762 | 51219 | 128135 |  | 3253607 |
| Grassland | 755528 |  | 4741267 | 506734 | 617360 | 2241091 |  | 8861980 |
| Cropland | 594548 | 1694671 |  | 70209 | 451866 | 364373 |  | 3175667 |
| Water | 21886 | 429813 | 142442 |  | 11163 | 381498 |  | 986802 |
| Built-up Land | 27834 | 229235 | 170900 | 6098 |  | 28712 |  | 462779 |
| Unused Land | 163005 | 4754029 | 1428254 | 501115 | 347500 |  |  | 7193903 |
| Unchanged |  |  |  |  |  |  | 1240080530 | 1240080530 |
| Total  Turning-in | 1562801 | 9524080 | 7117022 | 1107918 | 1479108 | 3143809 | 1240080530 | 1264015268 |

Table 10. The conversion of landscape classes in BM during 1990-2000 with pixel of unit (30 m*30 m).

|  | Forest | Grassland | Cropland | Water | Built-up Land | Unused Land | Unchanged | Total  Turning-out |
| --- | --- | --- | --- | --- | --- | --- | --- | --- |
| Forest |  | 1506192 | 849754 | 7114 | 11219 | 3147 |  | 2377426 |
| Grassland | 906880 |  | 2815193 | 11540 | 3043 | 3355 |  | 3740011 |
| Cropland | 218505 | 96337 |  | 45519 | 70859 | 129318 |  | 560538 |
| Water | 6220 | 77810 | 135178 |  | 395 | 1372 |  | 220975 |
| Built-up Land | 734 | 72 | 802 | 823 |  | 5 |  | 2436 |
| Unused Land | 4302 | 5439 | 1834231 | 16440 | 3 |  |  | 1860415 |
| Unchanged |  |  |  |  |  |  | 1260443540 | 1260443540 |
| Total  Turning-in | 1136641 | 1685850 | 5635158 | 81436 | 85519 | 137197 | 1260443540 | 1269205341 |

Table 11. The conversion of landscape classes in BM during 2000-2005 with pixel of unit (30 m*30 m).

|  | Forest | Grassland | Cropland | Water | Built-up Land | Unused Land | Unchanged | Total  Turning-out |
| --- | --- | --- | --- | --- | --- | --- | --- | --- |
| Forest |  | 624522 | 872012 | 61815 | 31168 | 18237 |  | 1607754 |
| Grassland | 847236 |  | 529374 | 29092 | 16248 | 75567 |  | 1497517 |
| Cropland | 941094 | 728085 |  | 70974 | 72983 | 17596 |  | 1830732 |
| Water | 20405 | 12323 | 22201 |  | 1207 | 114116 |  | 170252 |
| Built-up Land | 5629 | 3029 | 14780 | 1409 |  | 53 |  | 24900 |
| Unused Land | 26115 | 137486 | 345545 | 8808 | 244 |  |  | 518198 |
| Unchanged |  |  |  |  |  |  | 1263555428 | 1263555428 |
| Total  Turning-in | 1840479 | 1505445 | 1783912 | 172098 | 121850 | 225569 | 1263555428 | 1269204781 |

Table 12. The conversion of landscape classes in BM during 2005-2010 with pixel of unit (30 m*30 m).

|  | Forest | Grassland | Cropland | Water | Built-up Land | Unused Land | Unchanged | Total  Turning-out |
| --- | --- | --- | --- | --- | --- | --- | --- | --- |
| Forest |  | 16388177 | 4441387 | 1172181 | 151862 | 2267237 |  | 24420844 |
| Grassland | 21817430 |  | 1941998 | 21418895 | 96266 | 215902734 |  | 261177323 |
| Cropland | 2569323 | 2177471 |  | 728210 | 533916 | 1244202 |  | 7253122 |
| Water | 895913 | 3204537 | 179032 |  | 10417 | 5096350 |  | 9386249 |
| Built-up Land | 47667 | 23338 | 253948 | 14193 |  | 6571 |  | 345717 |
| Unused Land | 6448142 | 29324604 | 2126679 | 7798773 | 9123 |  |  | 45707321 |
| Unchanged |  |  |  |  |  |  | 920920536 | 920920536 |
| Total  Turning-in | 31778475 | 51118127 | 8943044 | 31132252 | 801584 | 224517094 | 920920536 | 1269211112 |

Table 13. The conversion of landscape classes in BM during 2010-2015 with pixel of unit (30 m*30 m).

|  | Forest | Grassland | Cropland | Water | Built-up Land | Unused Land | Unchanged | Total  Turning-out |
| --- | --- | --- | --- | --- | --- | --- | --- | --- |
| Forest |  | 1853153 | 1934697 | 207550 | 229612 | 444113 |  | 4669125 |
| Grassland | 1693188 |  | 2747014 | 865962 | 99299 | 2321122 |  | 7726585 |
| Cropland | 1704636 | 1328947 |  | 239281 | 415939 | 114244 |  | 3803047 |
| Water | 167004 | 550959 | 664934 |  | 8109 | 1787636 |  | 3178642 |
| Built-up Land | 31253 | 12612 | 104620 | 7085 |  | 531 |  | 156101 |
| Unused Land | 243554 | 2234830 | 2097334 | 2353174 | 8029 |  |  | 6936921 |
| Unchanged |  |  |  |  |  |  | 1243373891 | 1243373891 |
| Total  Turning-in | 3839635 | 5980501 | 7548599 | 3673052 | 760988 | 4667646 | 1243373891 | 1269844312 |

Table 14. The conversion of landscape classes in SWC during 1990-2000 with pixel of unit (30 m*30 m).

|  | Forest | Grassland | Cropland | Water | Built-up Land | Unused Land | Unchanged | Total  Turning-out |
| --- | --- | --- | --- | --- | --- | --- | --- | --- |
| Forest |  | 396584 | 290155 | 37157 | 34628 | 19845 |  | 778369 |
| Grassland | 356245 |  | 1551032 | 12834 | 10501 | 83454 |  | 2014066 |
| Cropland | 202986 | 83643 |  | 29837 | 121214 | 11105 |  | 448785 |
| Water | 2130 | 7827 | 63918 |  | 3721 | 1204 |  | 78800 |
| Built-up Land | 88 | 81 | 533 | 25 |  | 1 |  | 728 |
| Unused Land | 9044 | 46322 | 31383 | 1121 | 2 |  |  | 87872 |
| Unchanged |  |  |  |  |  |  | 269983138 | 269983138 |
| Total  Turning-in | 570493 | 534457 | 1937021 | 80974 | 170066 | 115609 | 269983138 | 273391758 |

Table 15. The conversion of landscape classes in SWC during 2000-2005 with pixel of unit (30 m*30 m).

|  | Forest | Grassland | Cropland | Water | Built-up Land | Unused Land | Unchanged | Total  Turning-out |
| --- | --- | --- | --- | --- | --- | --- | --- | --- |
| Forest |  | 148403 | 381647 | 66201 | 20107 | 767 |  | 617125 |
| Grassland | 962251 |  | 771230 | 16031 | 13492 | 86514 |  | 1849518 |
| Cropland | 1156027 | 1365143 |  | 53331 | 114269 | 127536 |  | 2816306 |
| Water | 7649 | 4960 | 15069 |  | 1301 | 779 |  | 29758 |
| Built-up Land | 3607 | 2877 | 10921 | 7534 |  | 63 |  | 25002 |
| Unused Land | 42291 | 71057 | 5355 | 3284 | 764 |  |  | 122751 |
| Unchanged |  |  |  |  |  |  | 267930746 | 267930746 |
| Total  Turning-in | 2171825 | 1592440 | 1184222 | 146381 | 149933 | 215659 | 267930746 | 273391206 |

Table 16. The conversion of landscape classes in SWC during 2005-2010 with pixel of unit (30 m*30 m).

|  | Forest | Grassland | Cropland | Water | Built-up Land | Unused Land | Unchanged | Total  Turning-out |
| --- | --- | --- | --- | --- | --- | --- | --- | --- |
| Forest |  | 861370 | 584444 | 182094 | 124243 | 3869 |  | 1756020 |
| Grassland | 691626 |  | 1515025 | 83940 | 147735 | 19212 |  | 2457538 |
| Cropland | 1618226 | 2821059 |  | 182574 | 619101 | 8999 |  | 5249959 |
| Water | 37379 | 26867 | 77944 |  | 19809 | 8156 |  | 170155 |
| Built-up Land | 15895 | 11042 | 78884 | 11957 |  | 294 |  | 118072 |
| Unused Land | 18522 | 131257 | 100460 | 5345 | 12337 |  |  | 267921 |
| Unchanged |  |  |  |  |  |  | 263374989 | 263374989 |
| Total  Turning-in | 2381648 | 3851595 | 2356757 | 465910 | 923225 | 40530 | 263374989 | 273394654 |

Table 17. The conversion of landscape classes in SWC during 2010-2015 with pixel of unit (30 m*30 m).

|  | Forest | Grassland | Cropland | Water | Built-up Land | Unused Land | Unchanged | Total  Turning-out |
| --- | --- | --- | --- | --- | --- | --- | --- | --- |
| Forest |  | 607087 | 950832 | 46722 | 214781 | 10255 |  | 1829677 |
| Grassland | 631913 |  | 2965612 | 52542 | 155922 | 93374 |  | 3899363 |
| Cropland | 951309 | 3422776 |  | 76394 | 497391 | 48421 |  | 4996291 |
| Water | 26497 | 29191 | 50043 |  | 11454 | 2728 |  | 119913 |
| Built-up Land | 34320 | 56680 | 218027 | 6917 |  | 3388 |  | 319332 |
| Unused Land | 20710 | 61408 | 37126 | 11150 | 10418 |  |  | 140812 |
| Unchanged |  |  |  |  |  |  | 262090414 | 262090414 |
| Total  Turning-in | 1664749 | 4177142 | 4221640 | 193725 | 889966 | 158166 | 262090414 | 273395802 |

Table 18. The conversion of landscape classes in WSC during 1990-2000 with pixel of unit (30 m*30 m).

|  | Forest | Grassland | Cropland | Water | Built-up Land | Unused Land | Unchanged | Total  Turning-out |
| --- | --- | --- | --- | --- | --- | --- | --- | --- |
| Forest |  | 3477221 | 9280400 | 44944 | 24094 | 290973 |  | 13117632 |
| Grassland | 1160711 |  | 4772596 | 209338 | 44204 | 1364968 |  | 7551817 |
| Cropland | 394998 | 506242 |  | 54255 | 168177 | 284124 |  | 1407796 |
| Water | 20062 | 170447 | 64374 |  | 737 | 147484 |  | 403104 |
| Built-up Land | 304 | 659 | 3302 | 13 |  | 1867 |  | 6145 |
| Unused Land | 13459 | 1553022 | 807859 | 254507 | 7823 |  |  | 2636670 |
| Unchanged |  |  |  |  |  |  | 1396418595 | 1396418595 |
| Total  Turning-in | 1589534 | 5707591 | 14928531 | 563057 | 245035 | 2089416 | 1396418595 | 1421541759 |

Table 19. The conversion of landscape classes in WSC during 2000-2005 with pixel of unit (30 m*30 m).

|  | Forest | Grassland | Cropland | Water | Built-up Land | Unused Land | Unchanged | Total  Turning-out |
| --- | --- | --- | --- | --- | --- | --- | --- | --- |
| Forest |  | 1278295 | 619023 | 31650 | 50133 | 150743 |  | 2129844 |
| Grassland | 1642640 |  | 697197 | 99719 | 23283 | 1282555 |  | 3745394 |
| Cropland | 525953 | 407529 |  | 13558 | 105515 | 75959 |  | 1128514 |
| Water | 23029 | 57249 | 24638 |  | 941 | 83102 |  | 188959 |
| Built-up Land | 7625 | 4764 | 24300 | 612 |  | 1356 |  | 38657 |
| Unused Land | 146414 | 628960 | 861004 | 185082 | 4857 |  |  | 1826317 |
| Unchanged |  |  |  |  |  |  | 1412483537 | 1412483537 |
| Total  Turning-in | 2345661 | 2376797 | 2226162 | 330621 | 184729 | 1593715 | 1412483537 | 1421541222 |

Table 20. The conversion of landscape classes in WSC during 2005-2010 with pixel of unit (30 m*30 m).

|  | Forest | Grassland | Cropland | Water | Built-up Land | Unused Land | Unchanged | Total  Turning-out |
| --- | --- | --- | --- | --- | --- | --- | --- | --- |
| Forest |  | 22691348 | 10028714 | 974204 | 342044 | 11371074 |  | 45407384 |
| Grassland | 22173114 |  | 5835599 | 1046493 | 372402 | 37809952 |  | 67237560 |
| Cropland | 7722262 | 5550942 |  | 686464 | 1460824 | 3593378 |  | 19013870 |
| Water | 481958 | 493100 | 532938 |  | 36028 | 879835 |  | 2423859 |
| Built-up Land | 133144 | 145623 | 1064441 | 40235 |  | 111743 |  | 1495186 |
| Unused Land | 3432386 | 38308605 | 3443526 | 2707335 | 173722 |  |  | 48065574 |
| Unchanged |  |  |  |  |  |  | 1237907761 | 1237907761 |
| Total  Turning-in | 33942864 | 67189618 | 20905218 | 5454731 | 2385020 | 53765982 | 1237907761 | 1421551194 |

Table 21. The conversion of landscape classes in WSC during 2010-2015 with pixel of unit (30 m*30 m).

|  | Forest | Grassland | Cropland | Water | Built-up Land | Unused Land | Unchanged | Total  Turning-out |
| --- | --- | --- | --- | --- | --- | --- | --- | --- |
| Forest |  | 2444553 | 3479852 | 196454 | 251430 | 5546127 |  | 11918416 |
| Grassland | 3888331 |  | 4281351 | 454233 | 313982 | 14819496 |  | 23757393 |
| Cropland | 1836882 | 952725 |  | 118705 | 691689 | 828901 |  | 4428902 |
| Water | 133229 | 284337 | 1067028 |  | 52671 | 780721 |  | 2317986 |
| Built-up Land | 48488 | 38922 | 262449 | 11105 |  | 43186 |  | 404150 |
| Unused Land | 1769217 | 5605147 | 3285610 | 958774 | 255066 |  |  | 11873814 |
| Unchanged |  |  |  |  |  |  | 1366879873 | 1366879873 |
| Total  Turning-in | 7676147 | 9325684 | 12376290 | 1739271 | 1564838 | 22018431 | 1366879873 | 1421580534 |

Table 22. The calculated results of selected landscape indices during 1990-2015 in WPSF.

| NP | 1990 | 2000 | 2005 | 2010 | 2015 |
| --- | --- | --- | --- | --- | --- |
| Built-up Land | 17571 | 17558 | 17693 | 20225 | 21007 |
| Cropland | 20349 | 22848 | 23992 | 16568 | 16109 |
| Forest | 20215 | 23203 | 23722 | 19301 | 19418 |
| Grassland | 28255 | 26176 | 27976 | 24669 | 24608 |
| Unused Land | 20063 | 18715 | 21052 | 19703 | 19571 |
| Water | 8740 | 8540 | 8841 | 10122 | 9836 |
| LPI | 1990 | 2000 | 2005 | 2010 | 2015 |
| Built-up Land | 0.462 | 0.8872 | 0.8873 | 1.154 | 1.3192 |
| Cropland | 67.9746 | 67.2646 | 68.6638 | 69.9381 | 69.3395 |
| Forest | 24.8867 | 25.0576 | 25.0466 | 21.6411 | 21.6785 |
| Grassland | 91.7707 | 90.8567 | 90.072 | 90.5959 | 90.6414 |
| Unused Land | 96.6905 | 96.6741 | 96.6676 | 90.9171 | 90.9078 |
| Water | 8.7103 | 8.7103 | 8.7103 | 7.7843 | 7.6401 |
| TE | 1990 | 2000 | 2005 | 2010 | 2015 |
| Built-up Land | 47956592.9 | 47731360.9 | 48424577.7 | 52876542.9 | 56613210.6 |
| Cropland | 284833369 | 311582246 | 319190127 | 297940266 | 299988986 |
| Forest | 165949681 | 180297435 | 184331094 | 167436949 | 166068612 |
| Grassland | 660907487 | 662001947 | 676759666 | 632236059 | 630006275 |
| Unused Land | 392026014 | 397808001 | 409714519 | 434327809 | 431811066 |
| Water | 95645513.2 | 105831064 | 107028516 | 140906611 | 141801651 |
| MN_AREA | 1990 | 2000 | 2005 | 2010 | 2015 |
| Built-up Land | 26.490347 | 26.4451643 | 26.6750683 | 25.7169011 | 29.1130045 |
| Cropland | 287.572462 | 284.277272 | 278.871436 | 427.532018 | 461.721012 |
| Forest | 145.420232 | 132.072447 | 129.26694 | 198.737773 | 189.690522 |
| Grassland | 1658.20708 | 1751.36316 | 1630.35196 | 1766.24235 | 1773.04506 |
| Unused Land | 2715.21971 | 2919.02253 | 2596.80546 | 2846.64804 | 2847.14179 |
| Water | 361.969448 | 380.738903 | 366.586305 | 262.622443 | 271.372721 |
| AM_SHAPE | 1990 | 2000 | 2005 | 2010 | 2015 |
| Built-up Land | 1.49908669 | 1.4545358 | 1.48725605 | 1.69959776 | 1.73101017 |
| Cropland | 5.89750963 | 6.0298806 | 6.26729998 | 6.85254992 | 6.81882657 |
| Forest | 3.59445166 | 3.64067883 | 3.70899077 | 4.02401527 | 4.03360131 |
| Grassland | 14.110527 | 14.0561841 | 13.988322 | 12.8520666 | 12.883472 |
| Unused Land | 9.35341613 | 9.58447446 | 9.18013979 | 8.35769204 | 8.38136242 |
| Water | 4.86482923 | 5.17202185 | 5.35746195 | 7.83944715 | 8.17286612 |
| COHESION | 1990 | 2000 | 2005 | 2010 | 2015 |
| Built-up Land | 94.9685125 | 94.9473573 | 95.0077801 | 95.1723799 | 95.7300562 |
| Cropland | 99.7256052 | 99.7267135 | 99.7252999 | 99.7740944 | 99.784219 |
| Forest | 99.4886998 | 99.4569756 | 99.4566858 | 99.5888009 | 99.553061 |
| Grassland | 99.9371513 | 99.9352934 | 99.9336692 | 99.9249087 | 99.9257716 |
| Unused Land | 99.9445027 | 99.9457329 | 99.9412757 | 99.9272289 | 99.9268573 |
| Water | 99.548387 | 99.5482672 | 99.5487268 | 99.5526373 | 99.5641615 |
| MESH | 1990 | 2000 | 2005 | 2010 | 2015 |
| Built-up Land | 0.38698338 | 0.40581161 | 0.48358401 | 4.15391885 | 12.5690251 |
| Cropland | 2768.70285 | 3025.85599 | 3105.28331 | 4174.32896 | 4797.52491 |
| Forest | 785.846351 | 792.733509 | 794.064478 | 1359.11252 | 1130.82605 |
| Grassland | 418353.596 | 409743.291 | 402121.948 | 341071.284 | 345669.233 |
| Unused Land | 1335335.81 | 1345070.88 | 1211964.16 | 1252232.04 | 1238568.97 |
| Water | 1506.09891 | 1405.70355 | 1430.54125 | 958.099011 | 986.505891 |

Table 23. The calculated results of selected landscape indices during 1990-2015 in BM.

| NP | 1990 | 2000 | 2005 | 2010 | 2015 |
| --- | --- | --- | --- | --- | --- |
| Built-up Land | 11021 | 11351 | 11555 | 13306 | 14693 |
| Cropland | 114149 | 117970 | 112720 | 114210 | 112619 |
| Forest | 51813 | 52639 | 52873 | 56523 | 55937 |
| Grassland | 44770 | 48199 | 47313 | 51281 | 50424 |
| Unused Land | 7757 | 9863 | 10333 | 15771 | 11453 |
| Water | 5104 | 6362 | 6356 | 9755 | 9920 |
| LPI | 1990 | 2000 | 2005 | 2010 | 2015 |
| Built-up Land | 0.2253 | 0.2764 | 0.327 | 0.5383 | 0.9232 |
| Cropland | 70.052 | 69.5858 | 69.2514 | 66.4169 | 82.8084 |
| Forest | 88.2976 | 87.9721 | 87.9775 | 88.1326 | 87.8722 |
| Grassland | 90.6065 | 90.6065 | 90.6035 | 76.8113 | 76.791 |
| Unused Land | 19.2205 | 18.9507 | 17.3271 | 60.7303 | 62.0675 |
| Water | 13.4975 | 14.3791 | 14.0443 | 13.5803 | 17.8434 |
| TE | 1990 | 2000 | 2005 | 2010 | 2015 |
| Built-up Land | 20950586.7 | 21538025.5 | 22689104.9 | 28016801.1 | 35778839.8 |
| Cropland | 729049415 | 738740826 | 750203052 | 758675498 | 734611450 |
| Forest | 963585128 | 1012799349 | 1028031976 | 1052925354 | 1058212506 |
| Grassland | 986508543 | 1012113401 | 1023058761 | 1127282261 | 1111177497 |
| Unused Land | 163305293 | 199647034 | 201413541 | 309396821 | 290653134 |
| Water | 45684311.5 | 61280307.3 | 62314817.1 | 126224451 | 129228081 |
| MN_AREA | 1990 | 2000 | 2005 | 2010 | 2015 |
| Built-up Land | 14.8167894 | 14.6230823 | 15.114957 | 16.2065059 | 18.3265744 |
| Cropland | 68.6072408 | 68.8217408 | 71.9903714 | 72.3948871 | 75.6683898 |
| Forest | 638.255533 | 623.741563 | 621.382284 | 589.409779 | 593.768746 |
| Grassland | 1016.27948 | 953.367188 | 971.238371 | 688.163334 | 694.666493 |
| Unused Land | 1183.36568 | 844.440109 | 803.48217 | 1096.47562 | 1491.18525 |
| Water | 422.153449 | 393.38996 | 393.78549 | 366.725967 | 365.147685 |
| AM_SHAPE | 1990 | 2000 | 2005 | 2010 | 2015 |
| Built-up Land | 1.05885979 | 1.09736967 | 1.12731281 | 1.52984573 | 1.76393965 |
| Cropland | 4.33693418 | 4.34109926 | 4.40915302 | 4.99250735 | 4.75378631 |
| Forest | 11.7816153 | 13.2831237 | 13.4449819 | 13.4863429 | 13.701505 |
| Grassland | 15.9544934 | 17.3631295 | 17.4694392 | 16.7709027 | 16.6465602 |
| Unused Land | 4.01727438 | 4.78506686 | 4.81331978 | 7.75457838 | 7.73165061 |
| Water | 3.81001908 | 4.37498626 | 4.73710665 | 6.27927893 | 6.55775005 |
| COHESION | 1990 | 2000 | 2005 | 2010 | 2015 |
| Built-up Land | 93.8086444 | 93.7998237 | 93.966411 | 94.4216603 | 95.2985317 |
| Cropland | 99.3385578 | 99.3505582 | 99.356148 | 99.4267753 | 99.4101312 |
| Forest | 99.872369 | 99.8859443 | 99.8857663 | 99.8749764 | 99.8762016 |
| Grassland | 99.9015732 | 99.9109511 | 99.9103984 | 99.8797166 | 99.8811777 |
| Unused Land | 99.7640801 | 99.7310545 | 99.729112 | 99.9070164 | 99.9126237 |
| Water | 99.3140577 | 99.494651 | 99.4973261 | 99.4793331 | 99.4914493 |
| MESH | 1990 | 2000 | 2005 | 2010 | 2015 |
| Built-up Land | 0.18142003 | 0.17563221 | 0.19771821 | 0.30541821 | 0.57361852 |
| Cropland | 4599.61496 | 5177.03612 | 5305.28723 | 5389.05881 | 9197.1863 |
| Forest | 191549.264 | 165040.828 | 164996.287 | 101065.292 | 102986.569 |
| Grassland | 1106422.71 | 1235715.8 | 1235778 | 144443.149 | 144507.846 |
| Unused Land | 18170.0098 | 5905.27041 | 5919.49947 | 138058.515 | 140799.012 |
| Water | 547.16485 | 865.188814 | 864.205931 | 974.374086 | 1049.06118 |

Table 24. The calculated results of selected landscape indices during 1990-2015 in SWC.

| NP | 1990 | 2000 | 2005 | 2010 | 2015 |
| --- | --- | --- | --- | --- | --- |
| Built-up Land | 13518 | 13772 | 13932 | 15770 | 16934 |
| Cropland | 87197 | 88391 | 84270 | 88976 | 90302 |
| Forest | 22270 | 22794 | 23506 | 24707 | 25065 |
| Grassland | 28025 | 28381 | 26036 | 27377 | 27586 |
| Unused Land | 1182 | 1203 | 1266 | 1317 | 1493 |
| Water | 3239 | 3195 | 2900 | 3295 | 3482 |
| LPI | 1990 | 2000 | 2005 | 2010 | 2015 |
| Built-up Land | 0.279 | 0.5175 | 0.5789 | 1.073 | 1.3375 |
| Cropland | 72.6159 | 72.5079 | 72.3401 | 71.6309 | 70.1866 |
| Forest | 86.7694 | 86.6848 | 86.2472 | 89.3475 | 89.3655 |
| Grassland | 70.9538 | 60.0076 | 59.3458 | 61.0964 | 61.1225 |
| Unused Land | 3.1568 | 3.1551 | 2.9073 | 2.0731 | 1.9214 |
| Water | 3.6035 | 3.5966 | 3.5981 | 3.5708 | 3.5718 |
| TE | 1990 | 2000 | 2005 | 2010 | 2015 |
| Built-up Land | 22261905.1 | 23457896.1 | 24697380.6 | 32438380.1 | 38607326 |
| Cropland | 772388119 | 780340519 | 783602109 | 750627062 | 754941177 |
| Forest | 362362590 | 366153256 | 385569901 | 380875315 | 385103601 |
| Grassland | 629203954 | 633875547 | 640328056 | 621447583 | 624184738 |
| Unused Land | 8712803.75 | 8852179.48 | 9727867.51 | 9307369.95 | 9957515.77 |
| Water | 21918187.5 | 21840245.6 | 22771118.8 | 23885800.1 | 24917926.4 |
| MN_AREA | 1990 | 2000 | 2005 | 2010 | 2015 |
| Built-up Land | 10.6729951 | 11.582669 | 12.2569661 | 15.4239653 | 17.394633 |
| Cropland | 83.6251867 | 84.0108275 | 86.3768709 | 78.8828263 | 76.9500413 |
| Forest | 432.574227 | 421.809497 | 414.988149 | 397.095104 | 390.804471 |
| Grassland | 256.621521 | 248.711157 | 270.227172 | 261.574393 | 260.480413 |
| Unused Land | 118.464347 | 118.467279 | 119.17368 | 99.007046 | 88.3703424 |
| Water | 61.4900741 | 62.3998317 | 72.3638271 | 71.7645817 | 69.7477669 |
| AM_SHAPE | 1990 | 2000 | 2005 | 2010 | 2015 |
| Built-up Land | 1.62989858 | 1.76725941 | 1.84014843 | 2.11797002 | 2.51354899 |
| Cropland | 12.5132011 | 12.479688 | 12.3239344 | 10.0805868 | 9.95192688 |
| Forest | 15.0067494 | 15.0683027 | 15.6036977 | 14.5056722 | 14.6149349 |
| Grassland | 22.260064 | 22.2781669 | 22.4997896 | 22.6840919 | 22.4372083 |
| Unused Land | 1.04000314 | 0.98355243 | 0.99407011 | 1.17309487 | 1.35530017 |
| Water | 5.85250872 | 5.84285897 | 6.28988029 | 6.22215443 | 6.24852492 |
| COHESION | 1990 | 2000 | 2005 | 2010 | 2015 |
| Built-up Land | 91.8666928 | 92.7935394 | 93.2425959 | 94.9615387 | 95.8600614 |
| Cropland | 99.468976 | 99.4676088 | 99.4430069 | 99.3189071 | 99.2991598 |
| Forest | 99.851131 | 99.8497728 | 99.844652 | 99.8381322 | 99.8376288 |
| Grassland | 99.7920939 | 99.7863794 | 99.7835179 | 99.7974715 | 99.7957205 |
| Unused Land | 98.802201 | 98.8114152 | 98.6388169 | 98.5121561 | 98.4574653 |
| Water | 98.4307591 | 98.4435945 | 98.5440047 | 98.6616648 | 98.6432468 |
| MESH | 1990 | 2000 | 2005 | 2010 | 2015 |
| Built-up Land | 0.22273972 | 0.57087503 | 0.72442559 | 2.17514564 | 3.75491235 |
| Cropland | 4320.90467 | 4430.20008 | 4130.39292 | 3020.63507 | 2951.21936 |
| Forest | 50726.7099 | 50261.12 | 50072.2385 | 46359.8672 | 46078.0055 |
| Grassland | 19963.609 | 16955.7885 | 16129.5027 | 17411.149 | 17315.5277 |
| Unused Land | 23.0686927 | 23.0782854 | 14.1913935 | 10.8584221 | 9.82626013 |
| Water | 14.6019369 | 15.3152816 | 18.1228697 | 25.4859816 | 25.6289117 |

Table 25. The calculated results of selected landscape indices during 1990-2015 in WSC.

| NP | 1990 | 2000 | 2005 | 2010 | 2015 |
| --- | --- | --- | --- | --- | --- |
| Built-up Land | 25014 | 25393 | 25689 | 30641 | 32132 |
| Cropland | 37052 | 40761 | 39668 | 43762 | 47037 |
| Forest | 52573 | 53729 | 54636 | 59376 | 58341 |
| Grassland | 43781 | 41932 | 44523 | 44403 | 43480 |
| Unused Land | 26821 | 26678 | 27170 | 22724 | 24749 |
| Water | 14235 | 14300 | 14112 | 16064 | 15826 |
| LPI | 1990 | 2000 | 2005 | 2010 | 2015 |
| Built-up Land | 9.0025 | 9.223 | 9.1935 | 11.6003 | 13.4135 |
| Cropland | 71.1253 | 71.1811 | 70.8451 | 47.2175 | 49.732 |
| Forest | 97.9036 | 95.7718 | 95.7641 | 94.2485 | 90.1024 |
| Grassland | 88.2509 | 88.2002 | 88.1867 | 92.7038 | 92.6873 |
| Unused Land | 74.927 | 74.9792 | 74.956 | 73.7114 | 74.0851 |
| Water | 16.5789 | 16.1626 | 16.1618 | 16.8829 | 16.8191 |
| TE | 1990 | 2000 | 2005 | 2010 | 2015 |
| Built-up Land | 52705796.8 | 54021955.2 | 55702301.7 | 67143051.2 | 75095362.1 |
| Cropland | 418333978 | 459496698 | 471630474 | 507869456 | 533008255 |
| Forest | 896542033 | 908775345 | 926491896 | 984770945 | 996153195 |
| Grassland | 1241886704 | 1237867520 | 1254698774 | 1121066677 | 1048274584 |
| Unused Land | 596759148 | 597822641 | 604021304 | 672541808 | 752319233 |
| Water | 155139942 | 156591648 | 158290472 | 189213621 | 189706573 |
| MN_AREA | 1990 | 2000 | 2005 | 2010 | 2015 |
| Built-up Land | 18.899405 | 19.4638812 | 19.7511536 | 19.1727502 | 21.5335184 |
| Cropland | 221.452989 | 231.149516 | 240.01084 | 221.448372 | 221.24443 |
| Forest | 826.938077 | 789.837447 | 777.086618 | 697.684989 | 703.505795 |
| Grassland | 1044.00871 | 1086.09049 | 1020.12281 | 1022.79944 | 1014.62333 |
| Unused Land | 1019.71566 | 1023.33908 | 1004.04306 | 1223.1244 | 1159.92733 |
| Water | 191.47547 | 191.610505 | 195.068185 | 188.336461 | 187.806491 |
| AM_SHAPE | 1990 | 2000 | 2005 | 2010 | 2015 |
| Built-up Land | 1.57324987 | 1.5809615 | 1.60844909 | 1.7341629 | 1.9456817 |
| Cropland | 4.86072101 | 5.11419968 | 5.18354132 | 5.15215439 | 5.42591269 |
| Forest | 12.6519975 | 12.473317 | 12.7577396 | 11.4358595 | 10.467784 |
| Grassland | 21.5608944 | 21.3832612 | 21.377988 | 16.7814164 | 16.5503961 |
| Unused Land | 11.2017516 | 11.2096237 | 11.3221453 | 12.2016251 | 12.6330833 |
| Water | 9.06702984 | 9.2419166 | 9.46320973 | 12.8558365 | 12.7571114 |
| COHESION | 1990 | 2000 | 2005 | 2010 | 2015 |
| Built-up Land | 94.657243 | 94.817617 | 94.8986663 | 95.0804565 | 95.7693881 |
| Cropland | 99.6379573 | 99.6578618 | 99.6569125 | 99.6131493 | 99.6450208 |
| Forest | 99.9270167 | 99.9238271 | 99.9242453 | 99.9067207 | 99.8933439 |
| Grassland | 99.9276638 | 99.9273982 | 99.9260226 | 99.9196237 | 99.9249203 |
| Unused Land | 99.9118218 | 99.9119782 | 99.9118205 | 99.8956969 | 99.8886032 |
| Water | 99.7756247 | 99.7713477 | 99.7707771 | 99.750931 | 99.7525506 |
| MESH | 1990 | 2000 | 2005 | 2010 | 2015 |
| Built-up Land | 0.68224551 | 0.8234287 | 0.85478968 | 1.04682709 | 1.87735047 |
| Cropland | 2499.996 | 3385.6606 | 3484.72137 | 2956.28984 | 4317.69868 |
| Forest | 277809.843 | 266335.901 | 270269.47 | 159734.321 | 106274.273 |
| Grassland | 272497.507 | 271520.397 | 266817.058 | 309582.414 | 311688.236 |
| Unused Land | 244342.317 | 243216.235 | 241700.631 | 219768.928 | 232460.227 |
| Water | 1477.07067 | 1433.46992 | 1420.31174 | 1530.41706 | 1556.80116 |
